# Construction of Phospholipid‐Like Vesicles by Aqueous Aminolysis of Acyl Thioesters With Diamino Acids

**DOI:** 10.1002/chem.202502881

**Published:** 2025-12-24

**Authors:** Federica A. Souto‐Trinei, Lucia Lomba‐Riego, Roberto J. Brea

**Affiliations:** ^1^ Bioinspired Nanochemistry (BioNanoChem) Group CICA ‐ Centro Interdisciplinar de Química e Bioloxía Departamento de Química, Facultad de Ciencias Universidade da Coruña A Coruña Spain

**Keywords:** aminolysis, lipid, membrane, self‐assembly, vesicle

## Abstract

Membrane‐forming phospholipids are produced in cells by enzymatic diacylation of polar head groups. However, analogous nonenzymatic processes remain both challenging and limited in scope. Moreover, the restricted variety of canonical lipid head groups constrains the properties and functions of phospholipid membranes. Therefore, the development of chemical methodologies for the straightforward preparation of versatile membrane‐forming building blocks is highly valuable. Here, we describe a simple strategy for the aqueous synthesis of phospholipid‐like molecules via direct aminolysis between diamino acids and water‐soluble acyl thioesters. In the presence of imidazole, diamino acids (i.e., diaminopropionic acid, ornithine, lysine) undergo rapid N‐diacylation to yield amphiphilic diacylated amino acids (DAAAs). These amino acid‐lipid conjugates spontaneously self‐assemble into robust micron‐sized vesicular structures capable of encapsulating hydrophilic cargos across a wide pH and temperature range. Our findings highlight the potential of amino acid‐based lipids as modular, functional building blocks for generating tunable membranous systems in aqueous environments.

## Graphical abstract

Membrane‐forming phospholipids are produced in cells via enzymatic diacylation. Here, we show that direct aminolysis of acyl thioesters with diamino acids in aqueous environments enables the synthesis of diacylated amino acids (DAAAs), which spontaneously self‐assemble into robust phospholipid‐like vesicles across a broad range of pH and temperature.

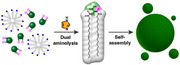

## 1 Introduction

Phospholipids, composed of a hydrophilic head group and two hydrophobic acyl chains, spontaneously organize into robust bilayer structures [1]. As the principal constituents of cellular membranes, their inherent amphiphilicity and self‐assembly behavior play an essential role in numerous practical applications such as the study of protein‐membrane interactions, drug delivery, origin‐of‐life research, and artificial reactors [2]. While the capability of phospholipids to self‐assemble into membranes is well characterized [3], their biosynthesis and membrane assembly from simple precursors are poorly understood. Moreover, the restricted number of canonical lipid head groups limits the properties and functions of phospholipid membranes [4, 5]. Therefore, one of the major goals of chemical biology is the development of simple and robust methods for the preparation of self‐assembling nonnatural membranes, which will help to understand the fundamental structural, dynamical, and biochemical features on which nature builds living systems [6].

The synthesis of phospholipids has become a powerful strategy in the creation of new bioactive materials, particularly in the design of carrier systems and the study of biological membranes [7, 8, 9]. Traditional methods for generating phospholipids often require the use of enzymes or complex reaction conditions [10, 11]. Over the last few years, the replication of this process from synthetic components has gained significant interest, as it offers more accessible, simplified, scalable, and sustainable routes for producing phospholipid‐like molecules [12, 13, 14, 15, 16]. Despite these advances, mimicking aqueous acylation processes found in natural systems remains challenging, mainly due to the need for specific functional groups, such as cysteines in native chemical ligation (NCL) [17, 18, 19] or histidines in histidine ligation [20]. Interestingly, recent work has shown that acylation in aqueous media can be achieved in the presence of simple reactive groups [14].

**FIGURE 1:**
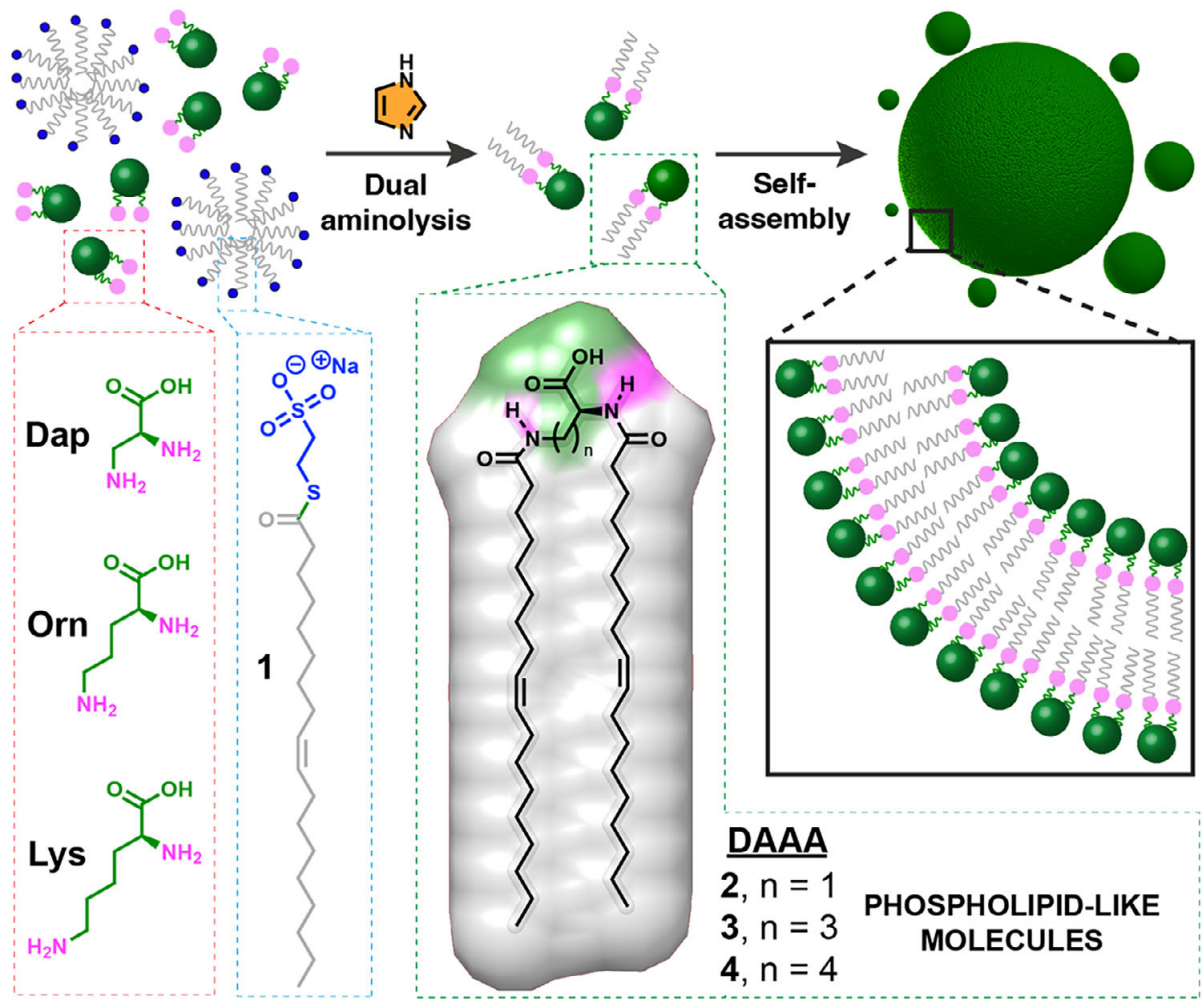
Schematic representation of spontaneous vesicle assembly induced by *in situ* synthesis of diacylated amino acids (DAAAs **2**, **3**, and **4**). The approach takes advantage of a dual imidazole‐promoted aminolysis ligation between a diamino acid (**Dap**, **Orn**, **Lys**) and an oleoyl thioester (**1**).

One of the main limitations of natural phospholipids is the low chemical versatility of their head groups, which restricts further functionalization and the introduction of new properties [21]. This constraint has motivated the development of alternative amphiphilic molecules that retain the self‐assembly and membrane‐forming features of canonical phospholipids but incorporate more readily modifiable polar head groups [9, 12, 22]. As a result, recent studies have emerged focusing on the design of phospholipid‐like molecules with unconventional head groups, aimed at expanding the functional landscape of lipid‐based materials and enabling new applications in biomedicine, sensing, and nanotechnology [13, 23, 24]. Among these, amino acids represent a particularly attractive class of polar head group analogues for the design of phospholipid mimics due to their special characteristics. Their natural abundance, biocompatibility, and low cost make them practical building blocks for the development of new biomolecules [3]. Importantly, amino acids possess a wide variety of structures, functional groups, and chirality, which allows for fine‐tuning of key properties such as charge, hydrophilicity, and the overall self‐assembly behavior of the resulting amphiphiles [25, 26, 27]. In addition to their structural versatility, amino acids are among the most fundamental building blocks of life and likely played a central role in early prebiotic chemistry [28]. Their spontaneous formation under plausible primitive conditions make them ideal candidates for studying the chemical pathways that could have led to the emergence of primitive membranes [29, 30]. Linking such prebiotic relevance with their synthetic utility highlights the potential of amino acid‐based systems to bridge the gap between simple molecular precursors and functional membrane‐forming amphiphiles. This versatility enables the creation of lipid analogues with tailored functionalities, expanding their potential for applications in biomimetic systems, targeted drug delivery, and synthetic biology [9, 19]. Furthermore, the use of amino acid‐based head groups facilitates the introduction of additional chemical handles for further modification, which can be used to incorporate targeting ligands [31], imaging agents [32], or responsive elements into lipid assemblies. Here, we describe the use of imidazole‐promoted reactions to rapidly prepare phospholipid‐like molecules via direct aminolysis ligations between diamino acids and fatty acyl thioesters (Figure 1). By using these new diamino building blocks, we aim to expand the chemical space of noncanonical amphiphiles and demonstrate the ease of generating functional phospholipid biomimetics that can spontaneously self‐assemble into stable micron‐sized vesicles with enhanced properties. These synthetic compartments could act as powerful platforms for the efficient encapsulation of relevant dyes and biomolecules.

## 2 Results and Discussion

### 2.1 Dual N‐acylation of Diamino Acids With Oleyl Thioesters

**FIGURE 2:**
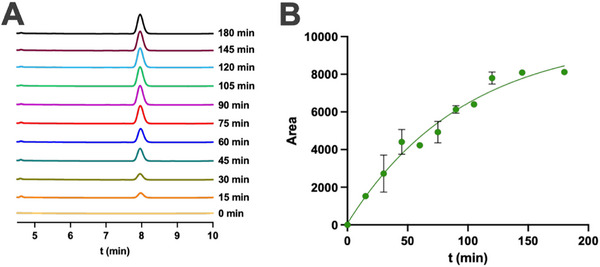
Synthesis of DAAA **2** by dual imidazole‐promoted aminolysis ligation between **Dap** and oleoyl thioester **1**. (A) Formation of DAAA **2** at various time points (0, 15, 30, 45, 60, 75, 90, 105, 120, 145, and 180 min). HPLC (210 nm) traces monitoring the dual aminolysis reaction between 25 mM **Dap** (1 equiv) and 50 mM oleoyl thioester **1** (2 equiv) in the presence of imidazole (1.5 M). Retention times (t_R_) were verified by mass spectrometry. (B) Kinetic plot of DAAA **2** formation during dual N‐acylation. Absorption at 210 nm was monitored and the area under the peak for compound **2** was plotted over time. Data was collected in triplicates.

We initially selected commercially available L‐2,3‐diaminopropionic acid (**Dap**) as a model diamino acid, given its compact structure and the spatial proximity of its two amino groups (Figure 1). This unique arrangement exhibits a close resemblance to the polar headgroup region of natural glycerophospholipids, making **Dap** a valuable starting point to explore site‐selective N‐acylation in water. As an acyl donor, we employed sodium 2‐mercaptoethanesulfonate (MESNA) thiooleate **1** [17], a stable (Figure S1), water‐soluble, and biocompatible thioester capable of undergoing aminolysis under mild conditions (Scheme S1) [14]. We next explored the aminolysis reactions between **Dap** and the previously prepared oleoyl thioester **1**, which could lead to the production of the diacylated amino acid (DAAA) **2** using millimolar concentrations of reactants (Figure 1). The generation of the amino acid‐lipid conjugate **2** was analyzed over time using high‐performance liquid chromatography (HPLC) and mass spectrometry (MS) measurements. As expected, no product formation was detected in the absence of an additive (Table 1, Entries 1 and 2), even after extended incubation times (20 h), both at room temperature (Table S1, Entry 1) and 37 °C (Table S1, Entry 2), highlighting the necessity of activation for efficient transformation. In contrast, the addition of imidazole as a nucleophilic catalyst significantly enhanced the reaction rate, resulting in good conversion in less than 2 h (Table 1, Entries 3 and 4). We monitored the reaction over a 3‐hour period and analyzed the mixture at various time points, consistently observing the exclusive formation of the DAAA product **2** at each interval (Figure 2). Interestingly, the monoacylated diamino acid (MAAA) intermediate was not observed during the reaction (Figure 2, Scheme S2). We believe these findings result from hydrophobic interactions between the alkyl chains of the MAAA and **1**, bringing them into close proximity, possibly within mixed micelles, and leading to a more rapid second acylation. Our data indicate that the formation of MAAA is the rate‐limiting step, and once generated, it quickly transforms into DAAA. It is worth noting that dual aminolysis carried out at 37 °C (Table 1, Entry 4) offers slightly higher conversions than the one performed at rt (Table 1, Entry 3). Minimal conversion improvements were observed upon prolonged reaction times (Table S1, Entries 3 and 4). Alternative catalytic approaches involving transition metal complexes (CuBr_2_, AgNO_3_), commonly used in direct aminolysis reactions [33, 34], were also explored; yet none of these additives afforded any product detection under the same aqueous conditions (Table 1, Entries 5–8; Table S1, Entries 5–8).

**TABLE 1:** Optimization study of the aqueous dual aminolysis reaction between diamino acids (**Dap**, **Orn**, **Lys**) and MESNA oleoyl thioester (**1**) after 2 h, affording the straightforward synthesis of DAAAs (**2**–**4**, respectively).[^a^]

Entry	Diamino acid	Additive	T (°C)	DAAA	Yield %
1	**Dap**		rt[^c^]	**2**	nd[^d^]
2	**Dap**		37	**2**	nd
3	**Dap**	Im[^b^] (1.5 M)	rt	**2**	57
4	**Dap**	Im (1.5 M)	37	**2**	70
5	**Dap**	CuBr_2_ (30 mM)	rt	**2**	nd
6	**Dap**	CuBr_2_ (30 mM)	37	**2**	nd
7	**Dap**	AgNO_3_ (30 mM)	rt	**2**	nd
8	**Dap**	AgNO_3_ (30 mM)	37	**2**	nd
9	**Orn**		rt	**3**	nd
10	**Orn**		37	**3**	nd
11	**Orn**	Im (1.5 M)	rt	**3**	55
12	**Orn**	Im (1.5 M)	37	**3**	67
13	**Orn**	CuBr_2_ (30 mM)	rt	**3**	nd
14	**Orn**	CuBr_2_ (30 mM)	37	**3**	nd
15	**Orn**	AgNO_3_ (30 mM)	rt	**3**	nd
16	**Orn**	AgNO_3_ (30 mM)	37	**3**	nd
17	**Lys**		rt[^a^]	**4**	nd
18	**Lys**		37	**4**	nd
19	**Lys**	Im (1.5 M)	rt	**4**	53
20	**Lys**	Im (1.5 M)	37	**4**	68
21	**Lys**	CuBr_2_ (30 mM)	rt	**4**	nd
22	**Lys**	CuBr_2_ (30 mM)	37	**4**	nd
23	**Lys**	AgNO_3_ (30 mM)	rt	**4**	nd
24	**Lys**	AgNO_3_ (30 mM)	37	**4**	nd

^a^ Conditions: Reaction mixture composed of 25 mM of diamino acid (1 equiv) and 50 mM of oleoyl thioester **1** (2 equiv), in the presence or absence of additive; 2 h of reaction.

^b^ Imidazole.

^c^ Room temperature (25 °C).

^d^ Not detected.

To assess how the spatial distribution of nucleophilic sites influences both reactivity and structural properties of the resulting phospholipid‐like molecules, we extended our study to include L‐ornithine (**Orn**) and L‐lysine (**Lys**) (Figure S2). These diamino acids incorporate additional methylene spacers in their side chains, introducing variable distances between the two potential sites of N‐acylation. This exploration is particularly interesting to understand how geometry and distance between the amino groups in the head group impact the formation of synthetic membranes, as well as their fluidity, packing, and transition temperatures. For each diamino acid, aminolysis reactions were carried out under aqueous conditions following a general procedure, obtaining DAAAs **3** and **4** by combining thioester **1** with **Orn** and **Lys**, respectively (Schemes S3 and S4). In the absence of any additive, the expected products were not detected (Table 1, Entries 9 and 10 for **Orn** and Entries 17 and 18 for **Lys**), even upon extended reaction times (Table S1, Entries 9 and 10 for **Orn**, Entries 17 and 18 for **Lys**). However, in the presence of imidazole, the desired amino acid‐lipid conjugates were obtained in good yield in less than 2 h (Table 1, Entries 11 and 12 for **Orn**, Entries 19 and 20 for **Lys**). Slightly better conversions were obtained at 37 °C (Table 1, Entry 12 for **Orn**, Entry 20 for **Lys**) than at rt (Table 1, Entry 11 for **Orn**, Entry 19 for **Lys**). However, extended incubation times did not show significant changes in the conversion yields (Table S1, Entries 11 and 12 for **Orn**, Entries 19 and 20 for **Lys**). As expected, metal‐promoted aminolysis did not afford the corresponding DAAAs (Table 1, Entries 13–16 for **Orn**, Entries 21–24 for **Lys**; Table S1, Entries 13–16 for **Orn**, Entries 21–24 for **Lys**).

Our results demonstrate that imidazole‐promoted aminolysis offers a robust and straightforward platform for the dual N‐acylation of diamino acids under aqueous conditions, providing an enzyme‐ and metal‐free route for the *in situ* synthesis of phospholipid‐like molecules.

### 2.2 Characterization of the DAAA Vesicular Structures

**FIGURE 3:**
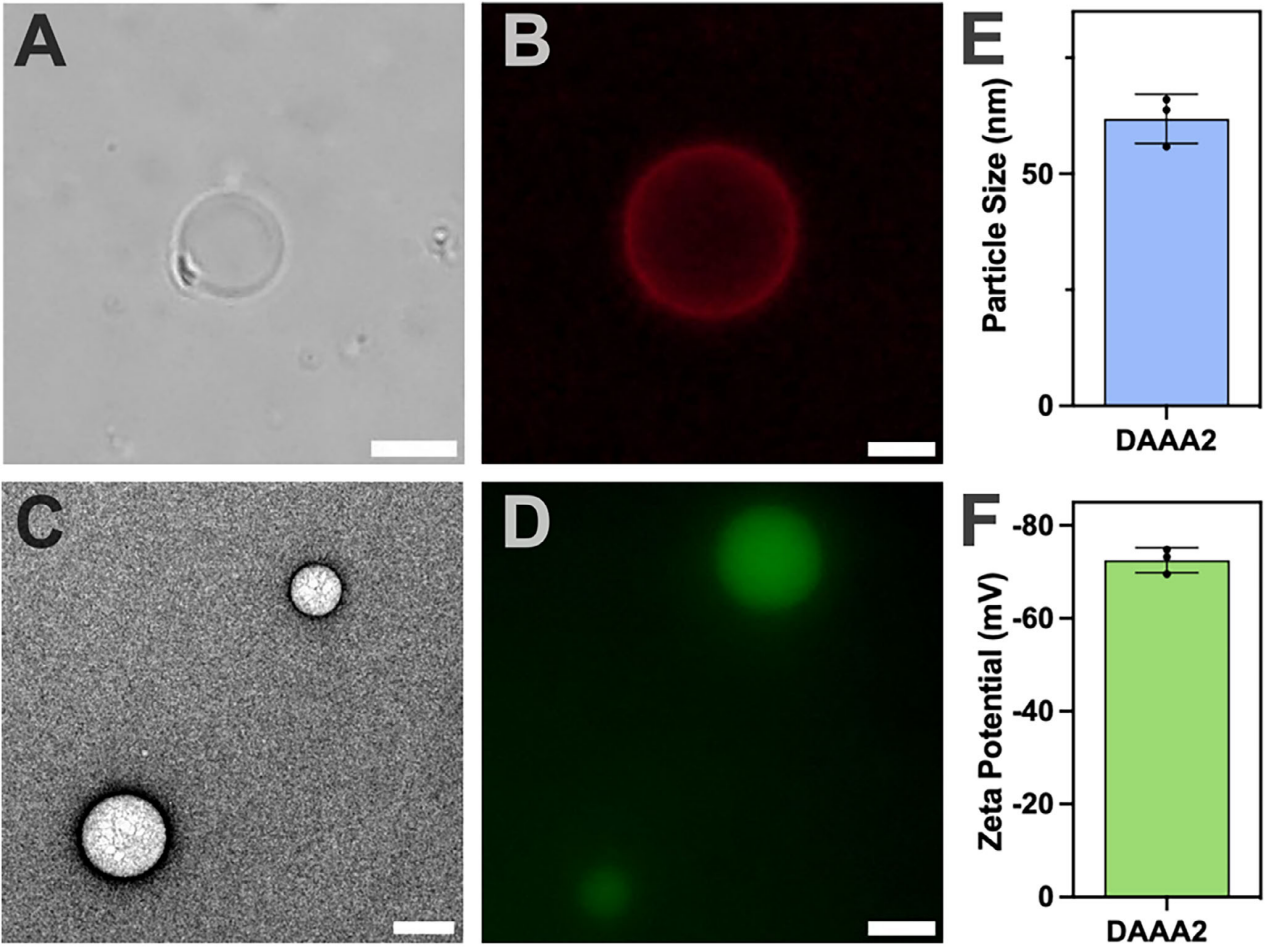
Characterization of DAAA **2** vesicle structures. (A) Phase contrast image of DAAA **2** vesicles. Scale bar denotes 5 µm. (B) Fluorescence microscopy image of vesicles resulting from the assembly of DAAA **2**. Membranes were stained using 0.1 mol% Nile Red dye. Scale bar denotes 5 µm. (C) TEM image of negatively stained **2** vesicles. Scale bar denotes 300 nm. (D) Fluorescence microscopy image demonstrating encapsulation of HPTS in **2** vesicles. Scale bar denotes 5 µm. (E) Particle size distribution determined by DLS of DAAA **2** vesicular nanoparticles obtained using the microfluidic‐based platform TAMARA. (F) Zeta potential measurements obtained by ELS of DAAA **2** vesicles generated using TAMARA.

We next explored the self‐assembly properties of the synthesized DAAAs through a comprehensive analysis of the morphology, size, surface charge, permeability, and stability of the obtained supramolecular structures. Hydrated samples of the purified compounds were first analyzed using phase contrast (Figure 3A, Figure S3) and fluorescence microscopy (Figure 3B, Figure S4). These preliminary observations revealed the spontaneous formation of well‐defined membrane‐bound assemblies (Figures 3A and 3B, Figures S3 and S4). Vesicle formation was consistently observed for all three DAAAs (**2**–**4**), indicating that structural differences in the amino acid side chains did not hinder their ability to self‐organize into micron‐sized vesicles in aqueous media. Negative‐staining transmission electron microscopy (TEM) also corroborated the formation of vesicular structures (Figure 3C, Figure S5). Encapsulation efficiency was evaluated using 8‐hydroxypyrene‐1,3,6‐trisulphonic acid (HPTS) as a fluorescent hydrophilic probe. Fluorescence microscopy confirmed successful loading of the dye within the corresponding DAAA vesicles (Figure 3D, Figure S6).

To gain further insights into the relationship between DAAA structure and vesicle formation, we prepared vesicular nanostructures for each amino acid‐lipid conjugate (**2**–**4**) using TAMARA, a microfluidic‐based platform that offers the precise generation of the corresponding lipid‐based nanoparticles. The size of the generated vesicles was characterized by dynamic light scattering (DLS), revealing a size distribution ranging from 60 to 76 nm (Figure 3E, Figure S7). The surface charge of the DAAA vesicles was determined to be anionic by zeta potential measurements obtained using electrophoretic light scattering (ELS), ranging from −72 to −50 mV (Figure 3F, Figure S7). Interestingly, we observed a consistent trend in both particle size and surface charge as a function of molecular structure. Specifically, as the overall size of the DAAA increased, the resulting nanoparticles exhibited larger hydrodynamic diameters and lower negative zeta potentials (Figure S7). This behavior suggests that either the increased distance between the hydrophobic tails, the elongation of the lipid tail or the presence of bulkier headgroups can affect the packing efficiency of the lipids within the membrane. Poorer packing likely leads to more loosely organized bilayers, which in turn give rise to vesicles of larger dimensions [35]. Additionally, the lower charge could be attributed to a reduced surface density of ionizable groups.

**FIGURE 4:**
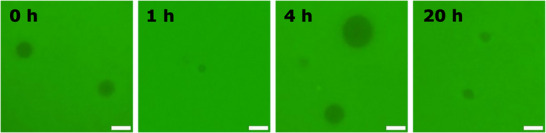
Fluorescence microscope images of DAAA **2** vesicles in 1 mM PBS (pH 7.0) at 37 °C and different time points (0, 1, 4, and 20 h) following the external addition of HPTS (50 µM). Scale bar denotes 5 µm.

We next evaluated the permeability of the DAAA vesicular architectures to charged small molecules. Permeability assays monitored by fluorescence microscopy showed that DAAA membranes were impermeable to externally added HPTS, further supporting the formation of intact bilayer structures with selective barrier properties, similar to those of biological membranes (Figure 4, Figure S8). Microscopic images were acquired from independent samples at each time point; therefore, the observed morphological differences arise from population heterogeneity rather than time‐dependent evolution of individual vesicles, consistent with the dynamic nature of these lipid assemblies.

**FIGURE 5:**
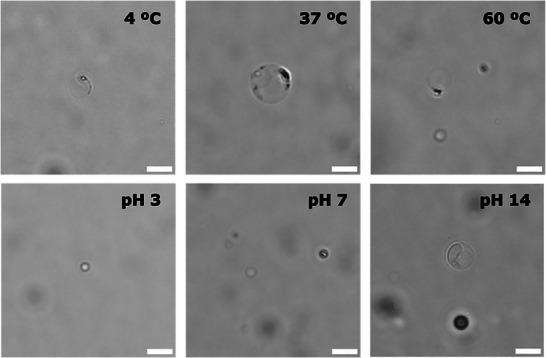
Representative phase‐contrast microscope images monitoring the stability of DAAA **2** vesicles taken after 20 h of incubation at different temperatures (4, 37, and 60 °C) and pH (3, 7, and 14). Scale bar denotes 5 µm.

We also assessed the robustness of the assemblies under different environmental conditions (Figure 5, Figures S9–S14). Vesicle samples of each DAAA were incubated for 20 h at various temperatures (4, 37, and 60 °C) and pH values (3, 7, and 14). In all cases, vesicle morphology and size distribution remained qualitatively comparable within experimental variability, highlighting the intrinsic stability of the DAAA membranes across a wide range of physiochemical conditions.

### 2.3 *In Situ* Formation of Vesicle Assemblies

**FIGURE 6:**
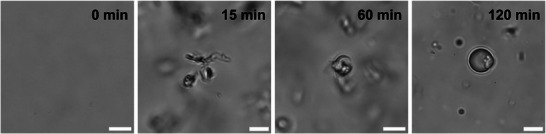
Phase‐contrast microscope images corresponding to *in situ* DAAA **2** vesicle formation. An aqueous solution of **Dap** (25 mM) and MESNA oleoyl thioester **1** (50 mM), in the presence of imidazole (1.5 M), was monitored at different time points. No DAAA membranes were observed immediately after combining both precursors. However, shortly after mixing, the spontaneous formation of vesicles and tubular structures was detected. After 2 h, large fields of vesicles were found. Scale bars denote 5 µm.

Once we demonstrated that the chemical *N*‐acylation of diamino acids occurs under physiological conditions, we next evaluated whether dual aminolysis and subsequent *de novo* vesicle assembly could happen spontaneously in water, omitting an intermediate purification step. The generation of the DAAA (**2**–**4**) vesicles was monitored by time‐lapse phase‐contrast microscopy (Figure 6, Figure S15). As expected, no visible aggregates were observed immediately after combining the diamino acid (**Dap**, **Orn**, or **Lys**; 25 mM) and **1** (50 mM) in the presence of imidazole (1.5 M). Shortly after mixing, tubular structures began forming, ultimately transforming into micron‐sized spherical vesicles. This *in situ* morphological change from tubular vesicles to spherical vesicles resembles earlier findings with amphiphilic molecules in aqueous solutions [17]. We additionally observed ongoing growth of vesicles, likely due to the persistent generation of **DAAA** within the bilayer of the vesicles formed *in situ*.

## 3 Conclusion

In summary, we have demonstrated that direct aminolysis can be employed as an enzyme‐free, robust, and versatile methodology for the *in situ* synthesis of functional DAAAs in aqueous media. These phospholipid‐like molecules self‐assemble into stable and organized vesicle structures over a wide temperature and pH range. By changing the spatial arrangement of these polar head groups, we obtained tunable membrane properties, including size and charge, while maintaining the same basic characteristics expected from natural membranes. We envision that the dual aminolysis approach can be applied for the *in situ* generation of diverse and biocompatible DAAA membranes, thus expanding the range of adaptable and programmable components in sophisticated membrane mimetics.

## 4 Experimental Section

### Materials and Methods

All reagents commercially supplied were used without further purification. Oleic acid, sodium 2‐mercaptoethanesulfonate (MESNA), 1‐(3‐dimethylaminopropyl)‐3‐ethylcarbodiimide hydrochloride (EDC·Cl), 3‐amino‐L‐alanine hydrochloride (H‐Dap‐OH·HCl), L‐ornithine monohydrochloride (H‐Orn‐OH·HCl), L‐lysine (Lys), imidazole (Im), and sodium 8‐hydroxypyrene‐1,3,6‐trisulfonate (HPTS) were purchased from BLD Pharmatech (China). 4‐Dimethylaminopyridine (DMAP) was purchased from Fluorochem (UK). Anhydrous dichloromethane (CH_2_Cl_2_) and trifluoroacetic acid (TFA) were purchased from Sigma‐Aldrich (USA). Deuterated solvents were purchased from Deutero GmbH (Germany). HPLC analysis was carried out on an Agilent 1260 Infinity II LC system (Agilent Technologies, USA) using an Eclipse Plus C8 analytical column with Phase A/Phase B gradients [Phase A: H_2_O with 0.1 % formic acid; Phase B: MeOH with 0.1 % formic acid]. HPLC purification was carried out on an Agilent 1260 Infinity II LC system (Agilent Technologies, USA) using a Zorbax SBC18 semipreparative column with Phase A/Phase B gradients [Phase A: H_2_O with 0.1 % formic acid; Phase B: MeOH with 0.1 % formic acid]. Proton nuclear magnetic resonance (^1^H‐NMR) spectra were recorded on a Bruker Avance AVA‐500 spectrometer (500 MHz) and were referenced relative to residual proton resonances in CD_3_OD (at 4.87 or 3.31 ppm) or d_6_‐DMSO (at 2.50 ppm). Chemical shifts were reported in parts per million (ppm, δ) relative to tetramethylsilane (δ 0.00). ^1^H NMR splitting patterns are assigned as singlet (s), doublet (d), triplet (t), quartet (q), or pentuplet (p). All first‐order splitting patterns were designated based on the appearance of the multiplet. Splitting patterns that could not be readily interpreted are designated as multiplet (m) or broad (br). Coupling constants are indicated in Hz. Carbon nuclear magnetic resonance (^13^C‐NMR) spectra were recorded on a Bruker Avance AVA‐500 spectrometer (500 MHz) and were referenced relative to residual carbon resonances in CD_3_OD (at 49.15 ppm) or d_6_‐DMSO (at 39.51 ppm). Electrospray ionization spectra (ESI‐Orbitrap‐MS) were obtained using a Thermo MAT95XP. Phase‐contrast microscopy images were obtained from an Olympus microscope model BX53, using a 100x oil immersion objective and a phase contrast condenser (PH3). Images were processed using Fiji. Transmission electron microscopy (TEM) images were recorded on a JEOL JEM‐1010 100 KV microscope equipped with a tungsten thermoionic electron gun, using the standard copper grids developed by Electron Microscopy Sciences. Dynamic Light Scattering (DLS) and Electrophoretic Light Scattering (ELS) were measured using a Malvern Zeta‐Sizer (Malvern Instruments, UK). Microfluidic experiments were performed using the TAMARA platform (Inside Therapeutics, France).

### Synthesis of Acyl Donors

*MESNA thiooleate* (**1**). A solution of oleic acid (189.2 mg, 670.0 µmol) in CH_2_Cl_2_ (5 mL) was stirred at 0°C for 10 min, and then DMAP (7.4 mg, 60.9 µmol) and EDC.HCl (128.4 mg, 670.0 µmol) were successively added. After 10 min stirring at 0°C, sodium 2‐mercaptoethanesulfonate (MESNA, 100.0 mg, 609.1 µmol) was added. After 5 h stirring at rt, the mixture was extracted with H_2_O (2 × 3 mL) and the combined aqueous phases were washed with EtOAc (3 mL). After evaporation of H_2_O under reduced pressure, the residue was washed with CH_3_CN (5 mL) and then filtered to yield 100.1 mg of **1** as a white solid (44 %). ^1^H‐NMR (d_6_‐DMSO, 500.13 MHz, δ): 5.36‐5.16 (m, 2H, 2× CH); 3.05‐2.85 (m, 2H, 1× CH_2_); 2.60‐2.51 (m, 4H, 2× CH_2_); 1.98‐1.94 (m, 4H, 2× CH_2_); 1.48 (q, *J* = 7,4 Hz, 2H, 2× CH_2_); 1.34‐1.08 (m, 24H, 12× CH_2_); 0.85 (t, *J* = 5.7 Hz, 3H, 1× CH_3_). ^13^C‐NMR (d_6_‐DMSO, 125.77 MHz, δ): 198.7; 129.8; 129.7; 51.0; 43.4; 31.4; 29.2; 29.1; 28.9; 28.8; 28.7; 28.6; 28.5; 28.3; 26.7; 26.6; 25.1; 24.4; 22.2; 14.1. HRMS (ESI) calculated for C_20_H_37_NaO_4_S_2_ [M‐Na]^−^: 405.2, found 405.2.

### Synthesis of DAAAs

*L‐2,3‐dioleoylpropanoic acid* (DAAA **2**). A solution of MESNA thiooleate (**1**) (61.3 mg, 143 µmol), H‐Dap‐OH·HCl (10.3 mg, 73.3 µmol) and imidazole (293.5 mg, 4311 µmol; Final concentration: 1.5 M) in H_2_O was stirred at rt for 2 h. The reaction was analyzed by HPLC, thus confirming product formation [t_R_ = 8.13 min (50–95 % Phase B in Phase A, 0 to 1 min; 95 % Phase B in Phase A, 1 to 8 min; 95–100 % Phase B in Phase A, 8 to 10 min; 100 % Phase B, 10 to 13 min; 100–50 % Phase B in Phase A, 13 to 15 min)]. Afterwards, the crude was purified by HPLC [t_R_ = 18.38 min (50–95 % Phase B in Phase A, 0 to 3 min; 95 % Phase B in Phase A, 3 to 5 min; 95–100 % Phase B in Phase A, 3 to 5 min; 100 % Phase B, 5 to 25 min; 100–50 % Phase B in Phase A, 25 to 27 min; 50 % Phase B in Phase A, 27 to 30 min)] to afford 26.4 mg of **2** as a colorless oil (57 %). ^1^H‐NMR (CD_3_OD, 500.13 MHz, δ): 5.40‐5.30 (m, 4H, 4× CH); 4.42 (t, *J* = Hz, 1H, 1× CH); 3.61 (dd, *J_1_* = 4.4 Hz, *J_2_* = 13.7 Hz, 1H, 1× CH); 3.52 (dd, *J_1_* = 8.0 Hz, *J_2_* = 13.7 Hz, 1H, 1× CH); 2.23 (t, *J* = 7.6 Hz, 2H, 1× CH_2_); 2.18 (t, *J* = 7.7 Hz, 2H, 1× CH_2_); 2.03 (q, *J* = 6.2 Hz, 8H, 4× CH_2_); 1.66–1.55 (m, 4H, 2× CH_2_); 1.41–1.24 (m, 40H, 20× CH_2_); 1.21–1.13 (m, 2H, 1× CH_2_); 0.91 (t, *J* = 6.8 Hz; 6H, 2× CH_3_). ^13^C‐NMR (CD_3_OD, 125.7 MHz, δ):177.5; 176.8; 175.9; 129.4; 40.5; 35.7; 31.7; 29.5; 29.4; 29.2; 29.1; 29.0; 28.9; 28.8; 26.8; 25.6; 25.4; 22.4; 13.1. LRMS (ESI) calculated for C_39_H_72_N_2_O_4_ [M+Na]^+^: 655.60, found 655.54.

*L‐2,5‐dioleoylpentanoic acid* (DAAA **3**). A solution of MESNA thiooleate (**1**) (50.8 mg, 118.61 µmol), H‐ornithine‐OH·HCl (10.0 mg, 59.3 µmol) and imidazole (237.4 mg, 3487 µmol; Final concentration: 1.5 M) in H_2_O was stirred at rt for 2 h. The reaction was analyzed by HPLC, thus confirming product formation [t_R_ = 7.31 min (50–95 % Phase B in Phase A, 0 to 1 min; 95 % Phase B in Phase A, 1 to 8 min; 95–100 % Phase B in Phase A, 8 to 10 min; 100 % Phase B, 10 to 13 min; 100–50 % Phase B in Phase A, 13 to 15 min)]. Afterwards, the crude was purified by HPLC [t_R_ = 17.33 min (50–95 % Phase B in Phase A, 0 to 3 min; 95 % Phase B in Phase A, 3 to 5 min; 95–100 % Phase B in Phase A, 3 to 5 min; 100 % Phase B, 5 to 25 min; 100–50 % Phase B in Phase A, 25 to 27 min; 50 % Phase B in Phase A, 27 to 30 min)] to afford 21.5 mg of **3** as a colorless oil (55 %). ^1^H‐NMR (CD_3_OD, 500.13 MHz, δ): 5.36–5.32 (m, 4H, 4× CH); 4.33 (t, *J* = 6.6 Hz, 1H, 1× CH); 3.17 (m, 2H, 1× CH_2_); 2.24 (t, *J* = 7.5 Hz, 1H, 1× CH); 2.16 (t, J = 7.5 Hz, 1H, 1× CH); 2.06‐1.99 (m, 8H, 4× CH_2_); 1.92–1.80 (m, 2H, 1× CH_2_); 1.70–1.50 (m, 6H, 3× CH_2_); 1.40–1.23 (m, 44H, 22× CH_2_); 0.89 (t, *J* = 6.9 Hz, 6H, 2× CH_3_). ^13^C‐NMR (CD_3_OD, 125.7 MHz, δ): 176.0; 175.6; 130.5; 57.3; 39.8; 37.0; 32.9; 30.7; 30.6; 30.4; 30.3; 30.2; 30.1; 28.0; 26.9; 26.8; 26.7; 23.6. LRMS (ESI) calculated for C_41_H_76_N_2_O_4_ [M+Na]^+^: 683.60, found 683.57.

*L‐2,6‐dioleoylhexanoic acid* (DAAA **4**). A solution of MESNA thiooleate (**1**) (61.6 mg, 143.65 µmol), L‐lysine (10.5 mg, 71.82 µmol) and imidazole (287.5 mg, 4223 µmol; Final concentration: 1.5 M) in H_2_O was stirred at rt for 2 h. The reaction was analyzed by HPLC, thus confirming product formation [t_R_ = 7.18 min (50–95 % Phase B in Phase A, 0 to 1 min; 95 % Phase B in Phase A, 1 to 8 min; 95–100 % Phase B in Phase A, 8 to 10 min; 100 % Phase B, 10 to 13 min; 100–50 % Phase B in Phase A, 13 to 15 min)]. Afterwards, the crude was purified by HPLC [t_R_ = 16.32 min (50–95 % Phase B in Phase A, 0 to 3 min; 95 % Phase B in Phase A, 3 to 5 min; 95–100 % Phase B in Phase A, 3 to 5 min; 100 % Phase B, 5 to 25 min; 100–50 % Phase B in Phase A, 25 to 27 min; 50 % Phase B in Phase A, 27 to 30 min)] to afford 25.6 mg of **4** as a colorless oil (53 %). ^1^H‐NMR (CD_3_OD, 500.13 MHz, δ): 5.50–5.29 (m, 4H, 4× CH); 4.35 (dd, *J_1_* = 9.0 Hz, *J_2_* = 4.7 Hz; 1H, 1× CH); 3.20–3.14 (m, 2H, 1× CH_2_); 2.24 (d, *J* = 7.7 Hz, 8H, 4× CH_2_); 2.16 (t, *J* = 7.4 Hz, 2H, 1× CH_2_); 2.06–1.99 (m, 8H, 4× CH_2_); 1.76–1.58 (m, 6H, 3× CH_2_); 1.34– 1.19 (m, 44H, 22× CH_2_); 0.90 (t, *J* = 6.9 Hz, 6H, 2× CH_3_). ^13^C‐NMR (CD_3_OD, 125.7 MHz, δ): 210.0; 176.2; 130.8; 53.8; 37.2; 33.1; 30.9; 30.9; 30.7; 30.5; 30.4; 30.4; 30.3; 30.3; 28.2; 27.0; 24.2; 23.8; 14.5. LRMS (ESI) calculated for C_42_H_78_N_2_O_4_ [M+Na]^+^: 697.60, found 697.54.

### Vesicle Formation

*Hydration method*. 10 µL of a 50 mM solution of DAAA (**2**–**4**) in a mixture 1:1 of MeOH:CHCl_3_ were added to a 1 mL glass vial and dried under N_2_ flow obtaining a lipid film. The corresponding lipid film was then hydrated using 100 µL of a 1 mM phosphate buffer (PBS) at pH 7.4 for 1 h at rt. Afterwards, the sample was analyzed by phase‐contrast microscopy, observing the formation of the expected vesicular architectures (Figure 3A, Figure S3). Vesicles were stained with a 0.1 mol% Nile Red (50 µM in EtOH) (Figure 3B, Figure S4).

*Microfluidics*. Vesicles were prepared by mixing an ethanolic solution of the DAAA (**2**–**4**) (Final concentration: 10 mg/mL) with a 1 mM PBS buffered (pH 7.4) solution in a ratio of 1:3 and a total flow rate of 4 mL/min. Generation of vesicular nanostructures was performed using TAMARA, a microfluidic‐based nanoparticle formulation system. DAAA nanoparticles (Final concentration of DAAA: 2.5 mg/mL) were collected in an Eppendorf tube. The size of the generated vesicles was characterized by dynamic light scattering (DLS) (Figure 3E, Figure S7), while the surface charge was determined by zeta potential analysis (Figure 3F, Figure S7).

*In situ vesicle formation*. In a 1.5 mL glass vial, an aqueous solution of the corresponding diamino acid (**Dap**, **Orn**, or **Lys**; 25 mM) was mixed with MESNA (50 mM) in the presence of imidazole (1.5 M). The mixture was stirred at rt. The progress of the vesicles formation was directly monitored without isolation or purification steps. At 0, 15, 60, and 120 min, aliquots were taken and observed under phase‐contrast microscopy (Figure 6, Figure S15).

### Transmission Electron Microscopy (TEM) Measurements

5 µL of a 2.5 mg/mL solution of DAAA (2, 3, or 4) vesicles in H_2_O (previously synthesized by using the TAMARA microfluidic platform) were deposited on the surface of a copper grid (formvar/carbon‐coated, 400 mesh copper). This solution was allowed to sit for 10 min before removing excess with filter paper and washing with 5 µL of filtered distilled water for 2 min. Excess was removed and samples were left drying for 30 min. Afterwards, samples were stained with 5 µL of 1 % w/w uranyl acetate. The stain was allowed to sit for 2 min before removing excess with filter paper. Then, it was washed with 5 µL of filtered distilled water. All grid treatments and simple depositions were on the dark/shiny/glossy formvar‐coated face of the grid. Samples were then imaged via TEM, revealing presence of several populations of spherical compartments of 50‐150 nm in diameter (Figure 3C, Figure S5).

### Dynamic Light Scattering (DLS) and Zeta Potential Measurements

DAAA (**2**–**4**) vesicle samples obtained by microfluidic formation (Final concentration of DAAA: 2.5 mg/mL) in 1 mM PBS buffered pH 7.4 solution were diluted (Final concentration: 0.25 mg/mL) with additional 1 mM PBS buffered pH 7.4 solution. The sample was then transferred to a DTS0012 cuvette Malvern Instruments to obtain size distribution (Figure 3E, Figure S7). Subsequently, the same sample was transferred to a DTS1070 Zetta potential cell to obtain zeta potential (Figure 3F, Figure S7).

### Encapsulation Studies

*Inverse‐emulsion method*. 1.2 µmol of DAAA (**2**‐**4**) were added to a 1 mL glass vial and dried under N_2_ flow until obtaining a lipid film. Then, 200 µL of mineral oil were added, and the mixture was sonicated for 1 h. Subsequently, 100 µL of the previous mixture were added to a 1 mL Eppendorf, to which was added 10 µL of upper buffer [50 µM HPTS + 200 mM sucrose in 100 mM HEPES buffer pH 7.5]. The resulting mixture was flicked until a cloudy emulsion was formed. The emulsion was then transferred to an Eppendorf containing 10 µL of lower buffer [200 mM glucose in 100 mM HEPES buffer pH 7.5], making sure the emulsion stayed on the upper phase. After 10 min, sample was centrifuged, and the mineral oil (top phase) was discarded. Lower phase contained the DAAA vesicles encapsulating the HPTS, which was analyzed by fluorescence microscopy (Figure 3D, Figure S6).

### Permeability Studies

0.5 µmol of DAAA (**2**–**4**) in a mixture 1:1 of MeOH:CHCl_3_ were added to a 1 mL glass vial and dried under N_2_ flow obtaining a lipid film. The corresponding lipid film was then hydrated using 100 µL of a 1 mM PBS (pH 7.4) buffered solution for 1 h at rt. Final lipid concentration was adjusted to 50 µM by diluting the sample with 1 mM PBS (pH 7.4) buffered solution containing 50 µM HPTS. Resulting samples were analyzed by fluorescence microscopy (Figure 4, Figure S8).

### Stability Studies

*Stability of MESNA thiooleate (* ***1*** *)*. A solution of MESNA thiooleate (**1**) (7.6 mg, 17.78 µmol) in H_2_O was stirred at rt for 20 h. The stability of **1** was analyzed by HPLC at different time points, observing that the thioester peak remained visible [t_R_ = 2.85 min (50‐95 % Phase B in Phase A, 0 to 3 min; 95 % Phase B in Phase A, 3 to 5 min; 95–100 % Phase B in Phase A, 3 to 5 min; 100 % Phase B, 5 to 25 min; 100–50 % Phase B in Phase A, 25 to 27 min; 50 % Phase B in Phase A, 27 to 30 min)] (Figure S1).

*Stability of DAAAs at different temperatures*. 0.5 µmol of DAAA (**2**–**4**) in a mixture 1:1 of MeOH:CHCl_3_ were added to a 1 mL glass vial and dried under N_2_ flow obtaining a lipid film. The corresponding lipid film was then hydrated using 100 µL of a 1 mM PBS (pH 7.4) buffered solution for 1 h at rt. Final lipid concentration was adjusted to 50 µM by diluting the sample with 1 mM PBS buffered (pH 7.4) solution. Resulting samples were incubated at different temperatures (4, 37, and 40 °C) for 20 h. Vesicular samples were then observed under phase‐contrast microscopy (Figure 5, Figures S9–S11). Samples were prepared under aqueous conditions without further purification, so minor debris or aggregates may appear in some micrographs.

*Stability of DAAAs at different pHs*. 0.5 µmol of DAAA (**2**–**4**) in a mixture 1:1 of MeOH:CHCl_3_ were added to a 1 mL glass vial and dried under N_2_ flow obtaining a lipid film. The corresponding lipid film was then hydrated using 100 µL of a 1 mM PBS (pH 7.4) buffered solution for 1 h at rt. Final lipid concentration was adjusted to 50 µM by diluting the sample with 1 mM PBS at different pHs (3.0, 7.0, and 14.0). Resulting samples were then incubated at different temperatures (4, 37, and 40 °C) for 20 h. Vesicular samples were then observed under phase‐contrast microscopy (Figure 5, Figures S12–S14). Samples were prepared under aqueous conditions without further purification, so minor debris or aggregates may appear in some micrographs.

## Conflicts of Interest

The author declares no conflict of interest.

## Supporting information



The authors have incorporated additional data within the Supporting Information, including Supplementary Schemes S1–S4, Supplementary Figures S1–S15, Supplementary Tables (Table S1) and NMR and MS spectra.
**Supporting File**: chem70617‐sup‐0001‐SuppMat.pdf.
