# Supplementary material for: Construction of Phospholipid‐Like Vesicles by Aqueous Aminolysis of Acyl Thioesters With Diamino Acids
Source: Chemistry. 2025 Dec 24;32(6):e02881. doi: 10.1002/chem.202502881 (PMC12887626; doi:10.1002/chem.202502881)
Supplement: Supplementary file 1 — The authors have incorporated additional data within the Supporting Information, including Supplementary Schemes S1–S4, Supplementary Figures S1–S15, Supplementary Tables (Table S1) and NMR and MS spectra. Supporting File: chem70617‐sup‐0001‐SuppMat.pdf. [file CHEM-32-e02881-s001.pdf]

Construction of Phospholipid-like Vesicles by Aqueous Aminolysis of Acyl Thioesters with Diamino Acids

Federica A. Souto-Trinei,[a] Lucia Lomba-Riego[a] and Roberto J. Brea*[a]

[a] Bioinspired Nanochemistry (BioNanoChem) Group
CICA - Centro Interdisciplinar de Química e Bioloxía
Departamento de Química, Facultad de Ciencias
Universidade da Coruña
Rúa As Carballeiras s/n, Campus de Elviña, 15071, A Coruña, Spain
E-mail: roberto.brea@udc.es
Homepage: https://bionanochemlab.com

SUPPLEMENTARY INFORMATION

Supplementary Schemes

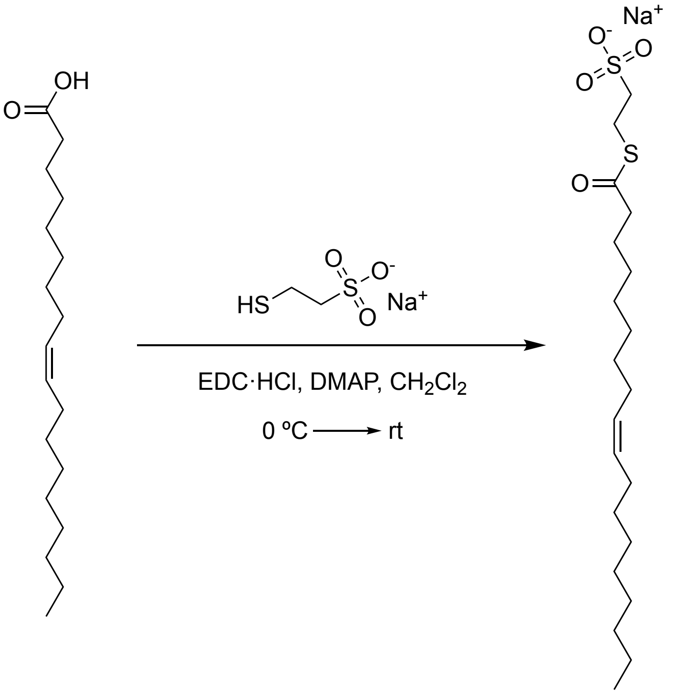

Scheme S1. Synthesis of MESNA thiooleate (**1**).

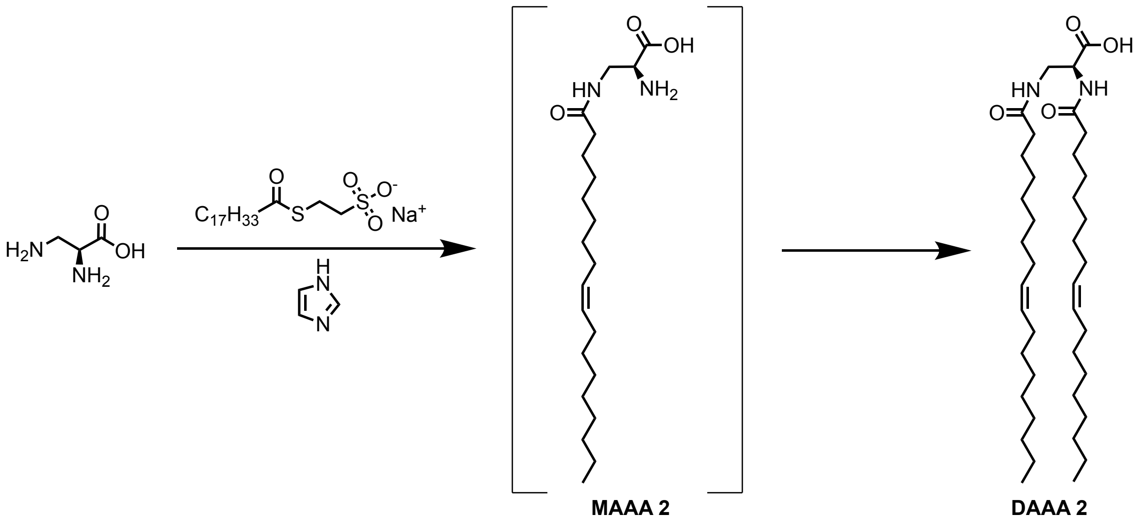

Scheme S2. Synthesis of L-2,3-dioleoylpropanoic acid (DAAA **2**). Intermediate *N*-3-oleoyl-L-2-aminopropanoic acid (MAAA **2**) is also shown.

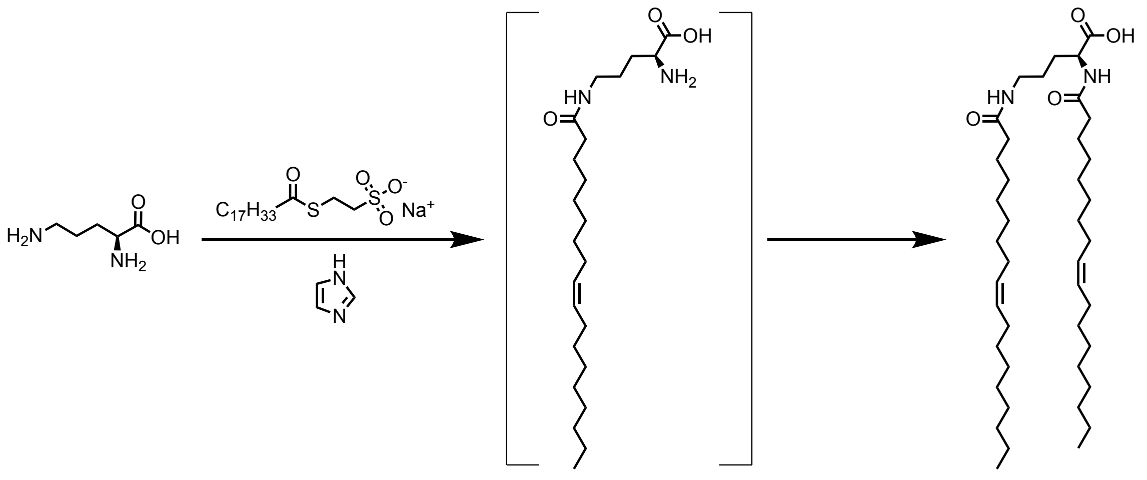

Scheme S3. Synthesis of L-2,5-dioleoylpentanoic acid (DAAA **3**). Intermediate *N*-5-oleoyl-L-2-aminopentanoic acid (MAAA **3**) is also shown.

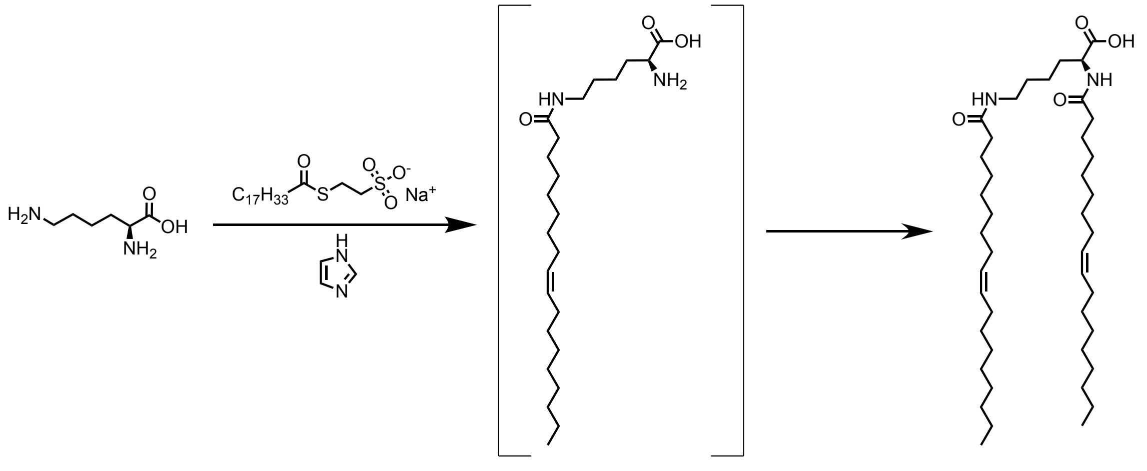

Scheme S4. Synthesis of L-2,6-dioleoylhexanoic acid (DAAA **4**). Intermediate *N*-6-oleoyl-L-2-aminohexanoic acid (MAAA **4**) is also shown.

Supplementary Figures

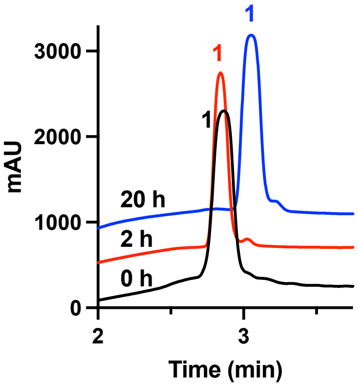

Figure S1. Stability analysis of oleyl thioester **1** in aqueous conditions at different time points. Retention times (t_R) were verified by mass spectrometry.

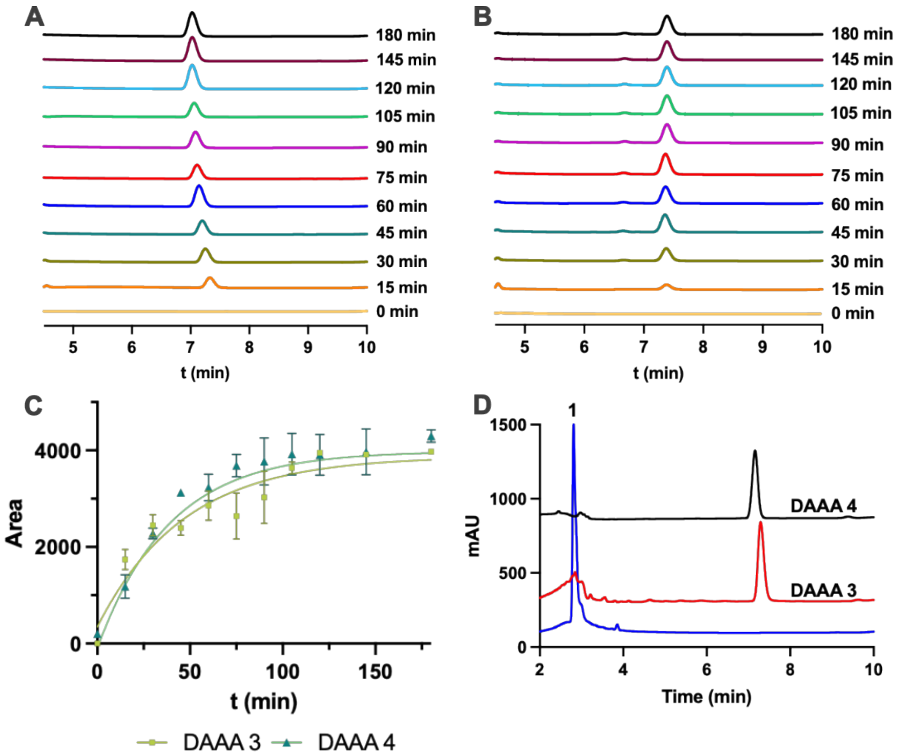

Figure S2. Formation of DAAA **3** (A) and **4** (B) at various time points (0, 15, 30, 45, 60, 75, 90, 105, 120, 145 and 180 min). HPLC (210 nm) traces monitoring the dual aminolysis reaction between 25 mM **Orn** or **Lys** (1 equiv) and 50 mM oleoyl thioester **1** (2 equiv) in the presence of imidazole (1.5 M). Retention times (t_R) were verified by mass spectrometry. C) Kinetic plots of DAAA **3** *(light green)* and **4** *(dark green)* formation during dual N-acylation. Absorption at 210 nm was monitored and the areas under the peaks for compound **3** and **4** were plotted over time. Data was collected in triplicates. D) HPLC (210 nm) traces corresponding to the dual aminolysis-based synthesis of DAAA **3** *(red)* and **4** *(black)* from the combination, in the presence of imidazole, of MESNA thioester **1** *(blue)* with **Orn** and **Lys**, respectively.

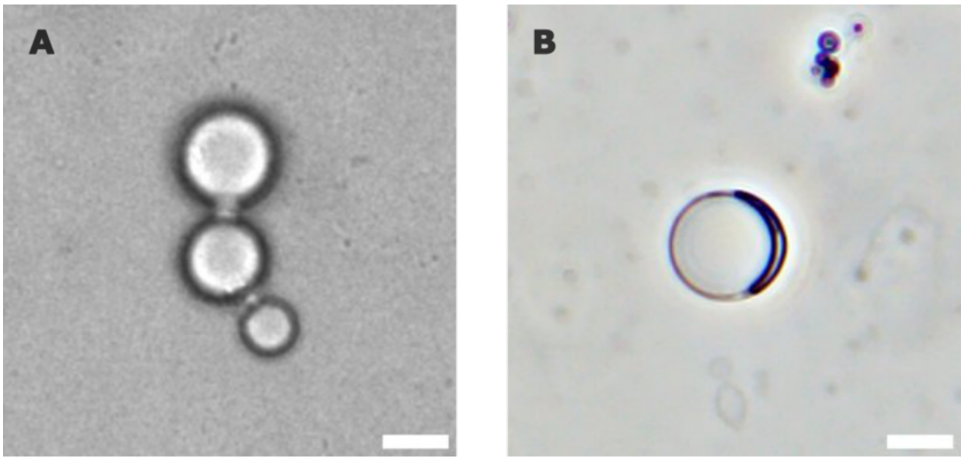

Figure S3. Phase-contrast microscopy images of DAAA vesicles resulting from the hydration in 1 mM phosphate saline buffer (pH 7.4) of lipid films of A) L-2,5-dioleoylpentanoic acid (**3**) and B) L-2,6-dioleoylhexanoic acid (**4**). Scale bars denote 5 μm.

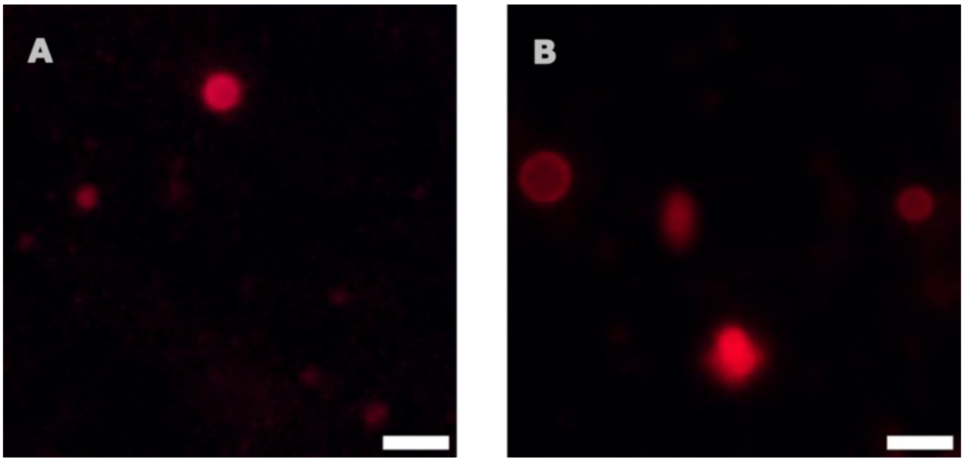

Figure S4. Fluorescence microscopy images of DAAA vesicles resulting from the assembly of A) L-2,5-dioleoylpentanoic acid (**3**) and B) L-2,6-dioleoylhexanoic acid (**4**). Membranes were stained using 0.1 mol% Nile Red dye. Scale bars denote 5 μm.

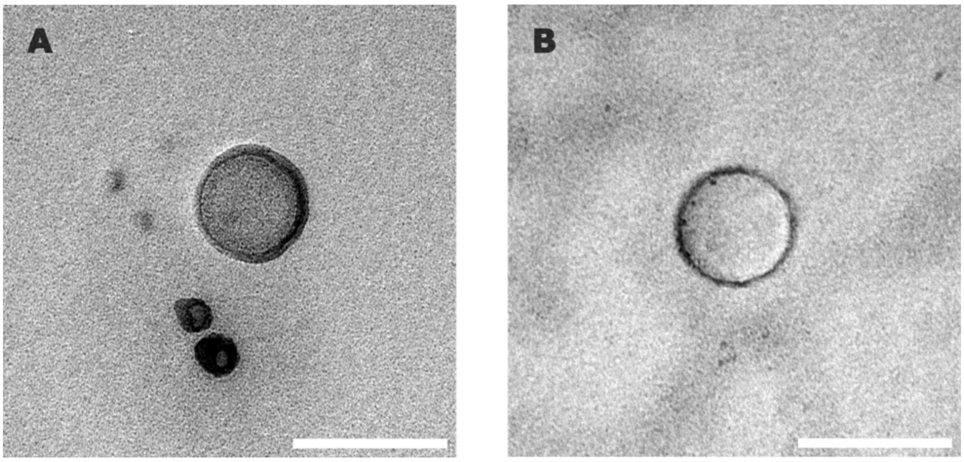

Figure S5. Transmission electron microscopy (TEM) images of negatively stained vesicles of A) L-2,5-dioleoylpentanoic acid (**3**) and B) L-2,6-dioleoylhexanoic acid (**4**). Scale bars denote 100 nm.

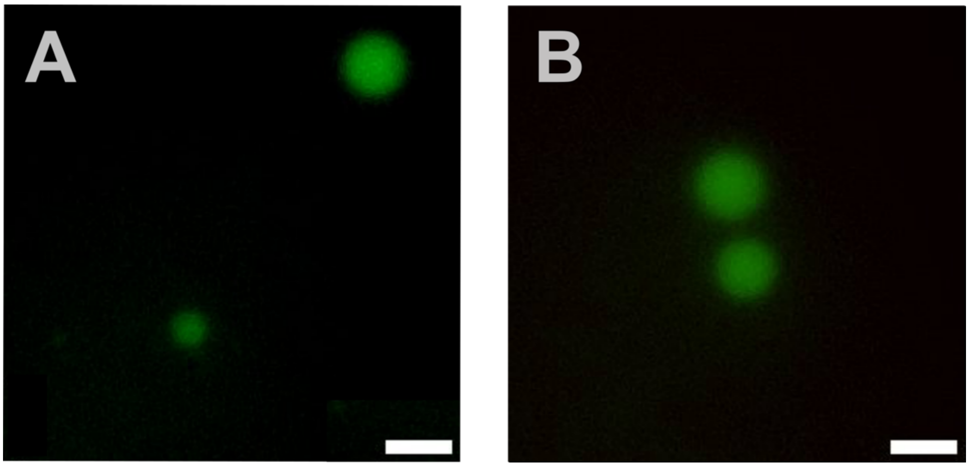

Figure S6. Fluorescence microscopy images demonstrating the encapsulation of HPTS in DAAA vesicles composed of A) L-2,5-dioleoylpentanoic acid (**3**) and B) L-2,6-dioleoylhexanoic acid (**4**). Scale bars denote 5 μm.

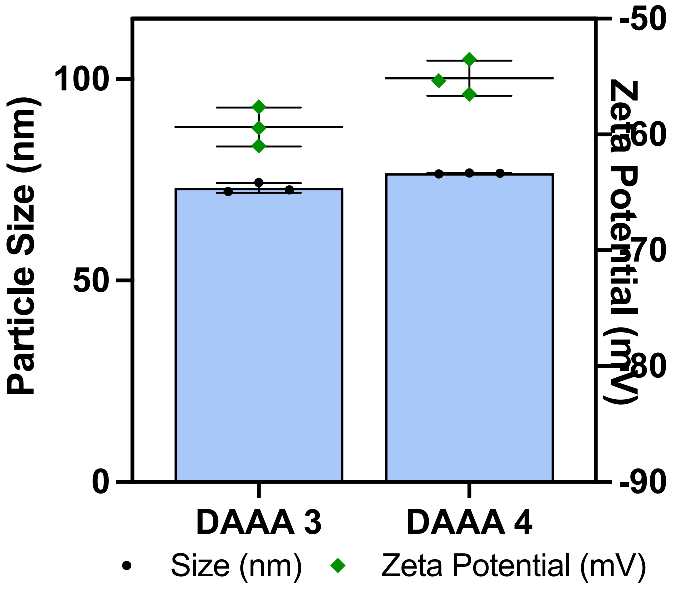

Figure S7. Particle size distribution *(left)* and zeta potential *(right)* of DAAA **3** and DAAA **4**, determined by dynamic light scattering (DLS) and electrophoretic light scattering (ELS), respectively.

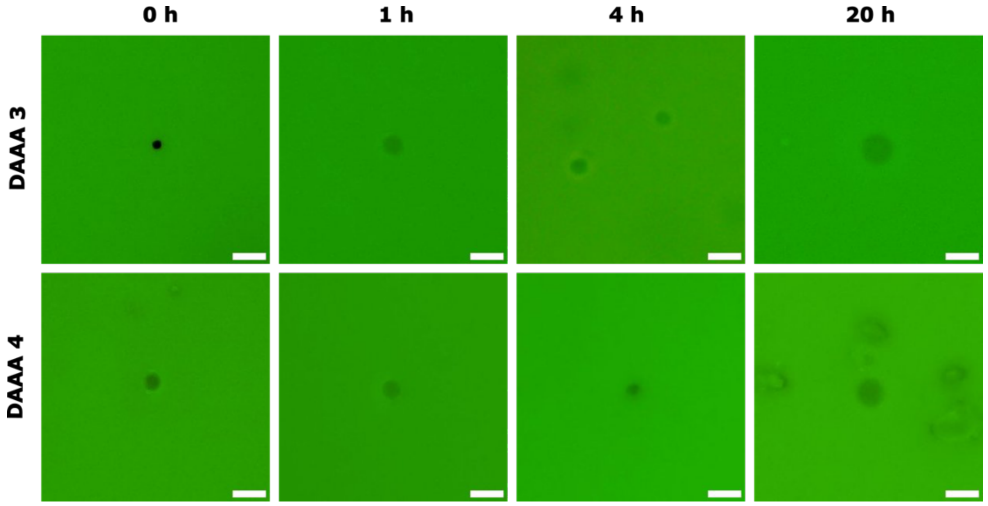

Figure S8. Fluorescence microscopy timelapse (0, 1, 2 and 4 h) images of vesicles of DAAA **3** and **4** in 1 mM PBS buffer (pH 7.4) at 37 ºC following the external addition of HPTS (50 µM). Scale bar denotes 5 µm.

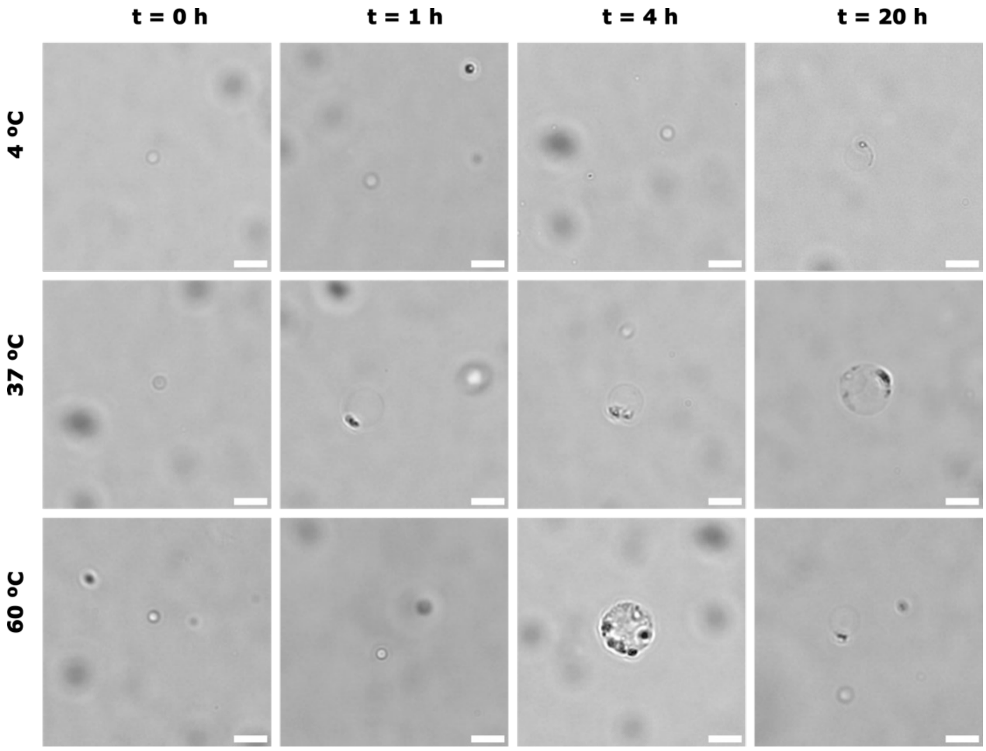

Figure S9. Representative phase-contrast microscopy timelapse (0, 1, 4 and 20 h) images of self-assembled vesicles of DAAA **2** in 1 mM PBS buffer (pH 7) at various temperatures (4, 37 and 60 ºC). Scale bars denote 5 µm.

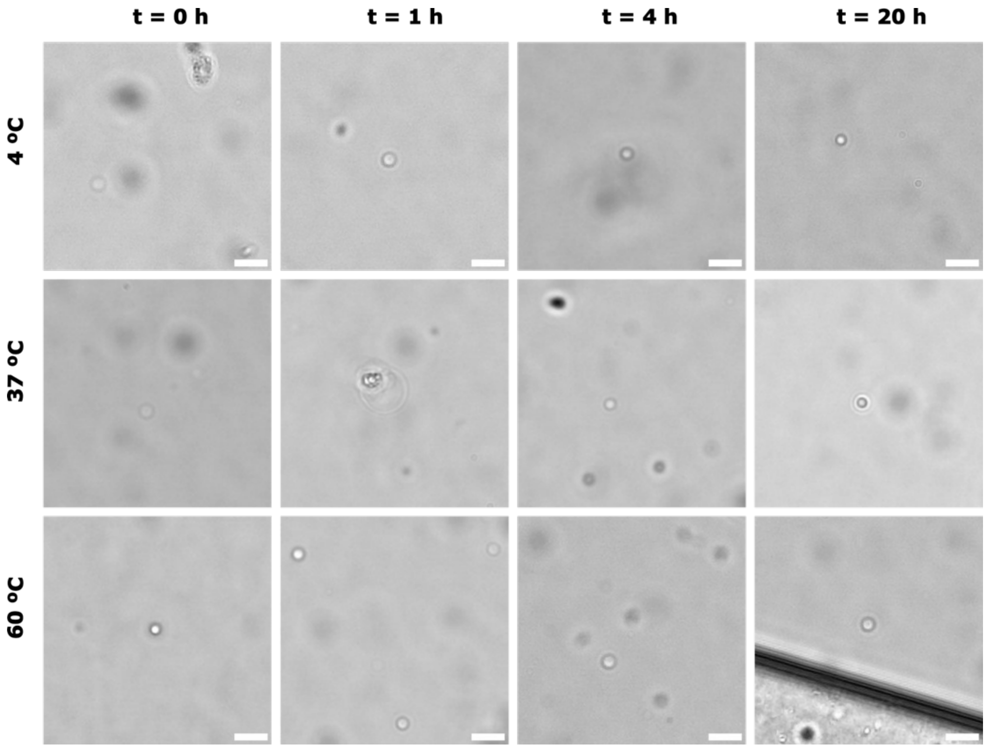

Figure S10. Representative phase-contrast microscopy timelapse (0, 1, 4 and 20 h) images of self-assembled vesicles of DAAA **3** in 1 mM PBS buffer (pH 7) at various temperatures (4, 37 and 60 ºC). Scale bars denote 5 µm.

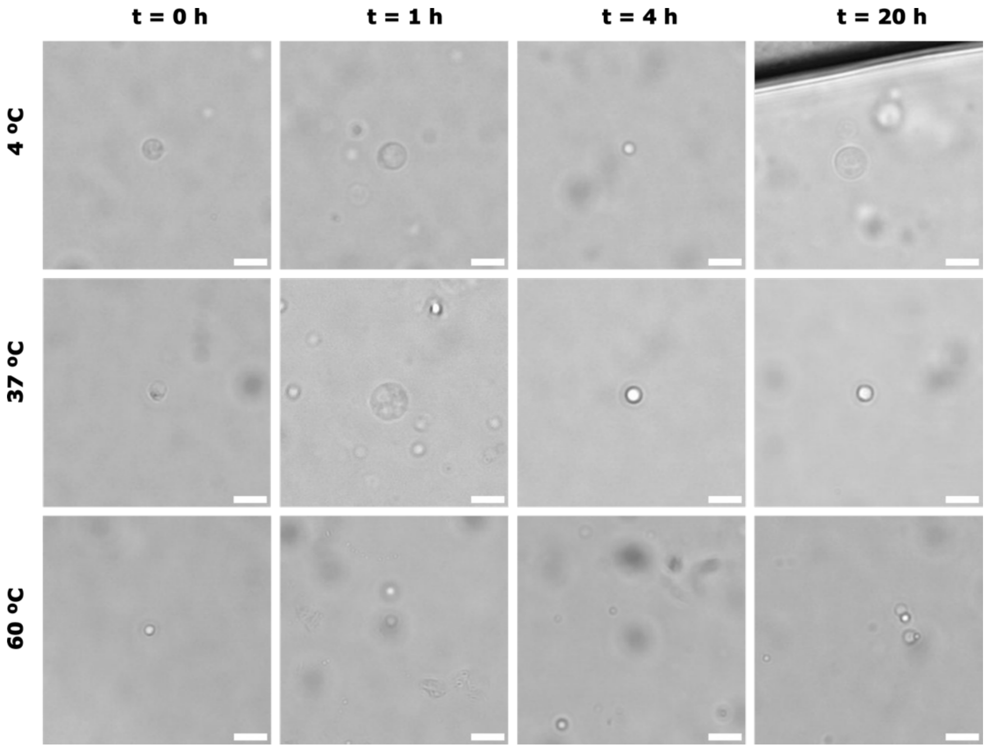

Figure S11. Representative phase-contrast microscopy timelapse (0, 1, 4 and 20 h) images of self-assembled vesicles of DAAA **4** in 1 mM PBS buffer (pH 7) at various temperatures (4, 37 and 60 ºC). Scale bars denote 5 µm.

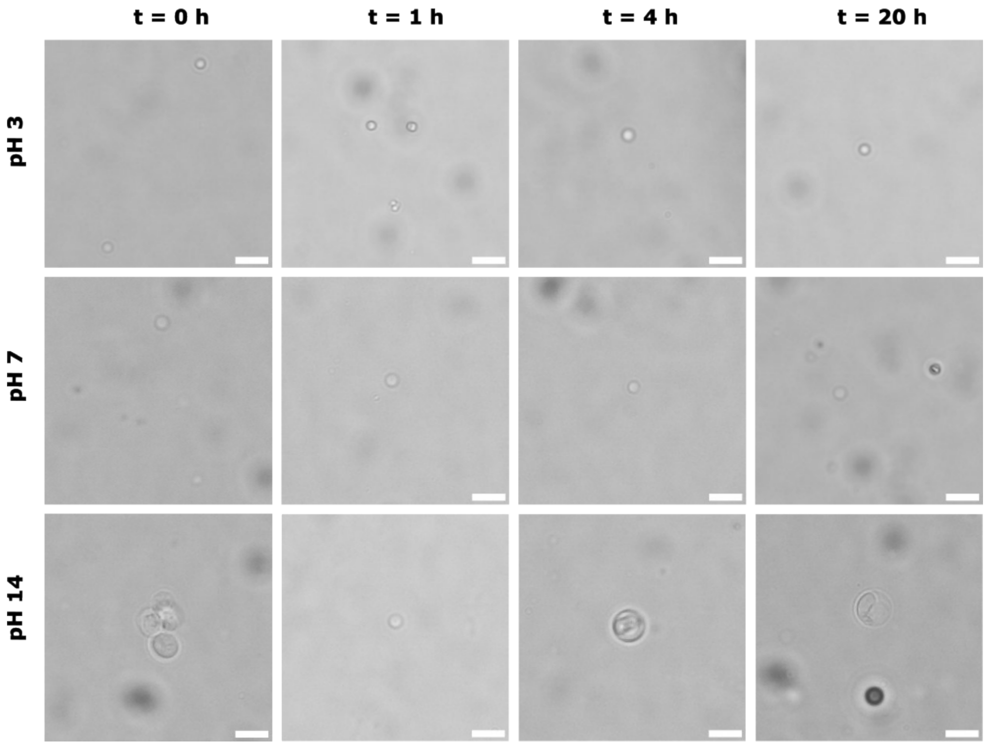

Figure S12. Representative phase-contrast microscopy timelapse (0, 1, 4 and 20 h) images of self-assembled vesicles of DAAA **2** in 1 mM PBS buffer (pH 7) at various pHs (3, 7 and 14). Scale bars denote 5 µm.

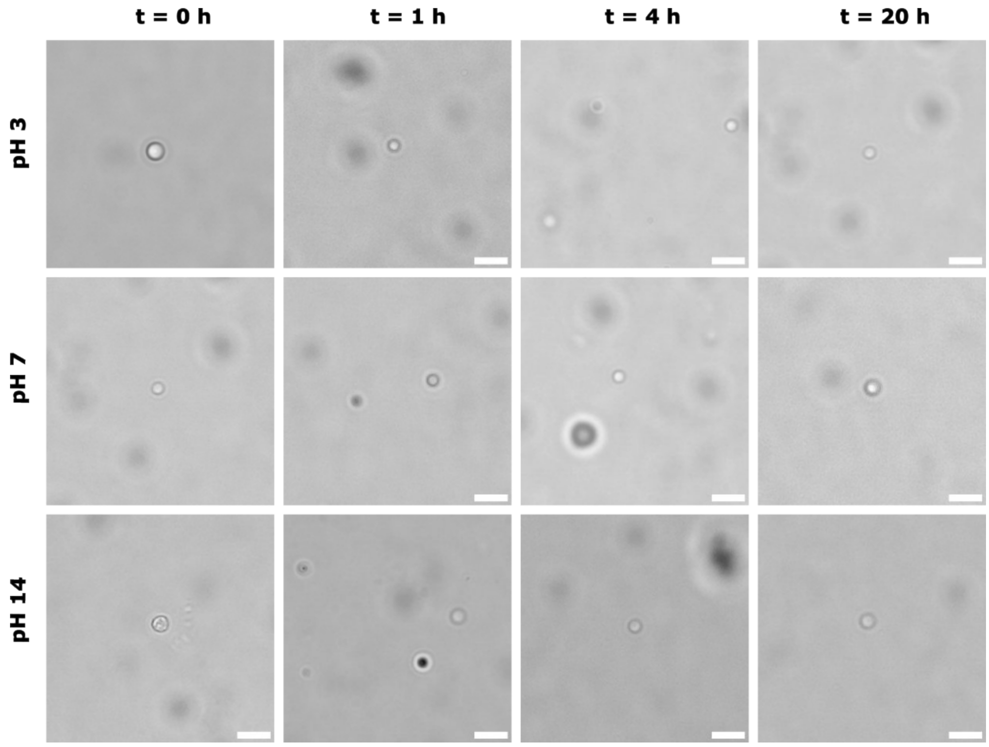

Figure S13. Representative phase-contrast microscopy timelapse (0, 1, 4 and 20 h) images of self-assembled vesicles of DAAA **3** in 1 mM PBS buffer (pH 7) at various pHs (3, 7 and 14). Scale bars denote 5 µm.

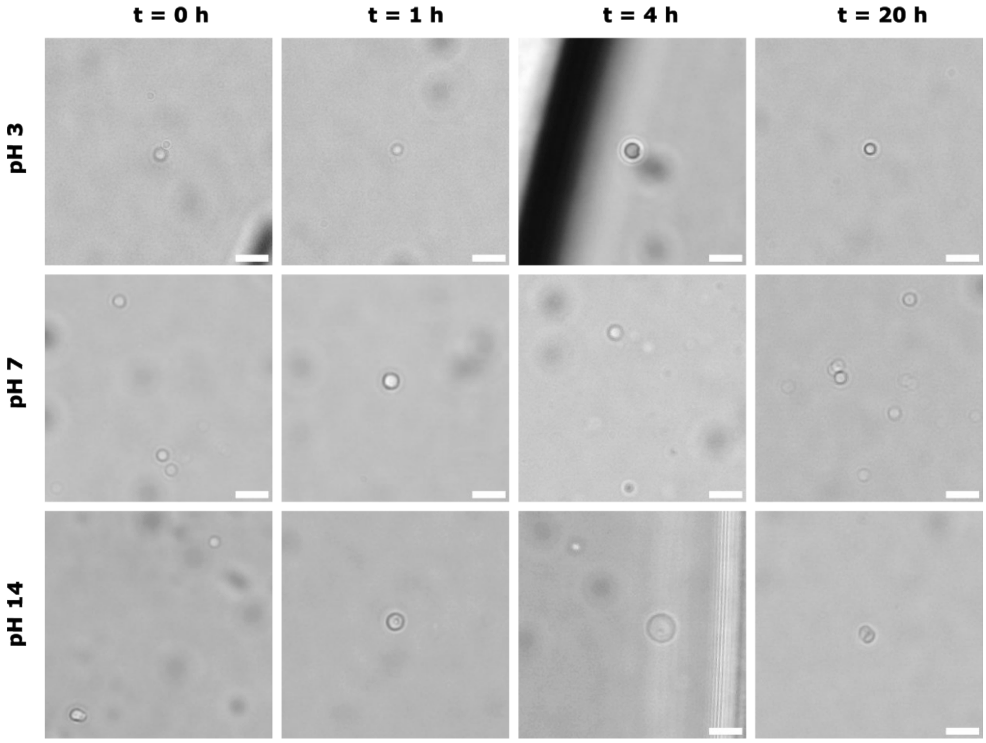

Figure S14. Representative phase-contrast microscopy timelapse (0, 1, 4 and 20 h) images of self-assembled vesicles of DAAA **4** in 1 mM PBS buffer (pH 7.4) at various pHs (3, 7 and 14). Scale bars denote 5 µm.

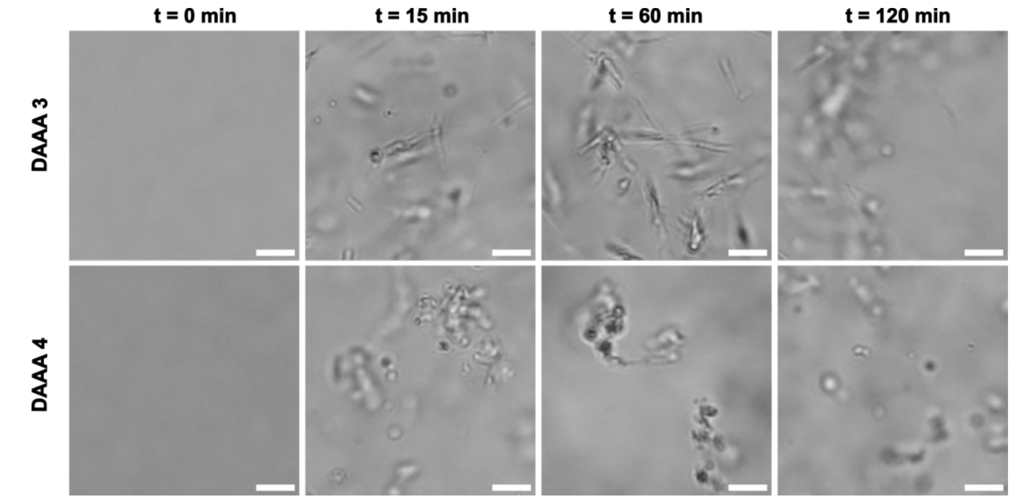

Figure S15. Phase contrast microscopy images corresponding to in situ DAAA **3** and DAAA **4** vesicle formation. An aqueous solution of the amino acid (**Orn** or **Lys**; 25 mM) and the MESNA thiooleate (**1**; 50 mM) in the presence of imidazole (1.5 M) was monitored at different time points (0, 15, 60 and 120 min). Scale bar denotes 5 μm.

Supplementary Tables

Table S1. Additional optimization study of the aqueous dual aminolysis reaction between diamino acids (**Dap**, **Orn**, **Lys**) and MESNA oleoyl thioester (**1**), affording the straightforward synthesis of DAAAs (**2-4**, respectively).[a]

Entry	Diamino acid	Additive	T (ºC)	DAAA	Yield %
1	**Dap**		rt[c]	**2**	nd[d]
2	**Dap**		37	**2**	nd
3	**Dap**	Im[b] (1.5M)	rt	**2**	55
4	**Dap**	Im (1.5M)	37	**2**	72
5	**Dap**	$CuBr_2$ (30 mM)	rt	**2**	nd
6	**Dap**	$CuBr_2$ (30 mM)	37	**2**	nd
7	**Dap**	$AgNO_3$ (30 mM)	rt	**2**	nd
8	**Dap**	$AgNO_3$ (30 mM)	37	**2**	nd
9	**Orn**		rt	**3**	nd
10	**Orn**		37	**3**	nd
11	**Orn**	Im (1.5M)	rt	**3**	56
12	**Orn**	Im (1.5M)	37	**3**	69
13	**Orn**	$CuBr_2$ (30 mM)	rt	**3**	nd
14	**Orn**	$CuBr_2$ (30 mM)	37	**3**	nd
15	**Orn**	$AgNO_3$ (30 mM)	rt	**3**	nd
16	**Orn**	$AgNO_3$ (30 mM)	37	**3**	nd
17	**Lys**		rt	**4**	nd
18	**Lys**		37	**4**	nd
19	**Lys**	Im (1.5M)	rt	**4**	54
20	**Lys**	Im (1.5M)	37	**4**	68
21	**Lys**	$CuBr_2$ (30 mM)	rt	**4**	nd
22	**Lys**	$CuBr_2$ (30 mM)	37	**4**	nd
23	**Lys**	$AgNO_3$ (30 mM)	rt	**4**	nd
24	**Lys**	$AgNO_3$ (30 mM)	37	**4**	nd

[a] Conditions: Reaction mixture composed of 25 mM of diamino acid (1 equiv) and 50 mM of oleoyl thioester **1** (2 equiv), in the presence or absence of additive; 20 h of reaction.

[b] Imidazole.

[c] Room temperature (25 ºC).

[d] Not detected.

NMR Spectra

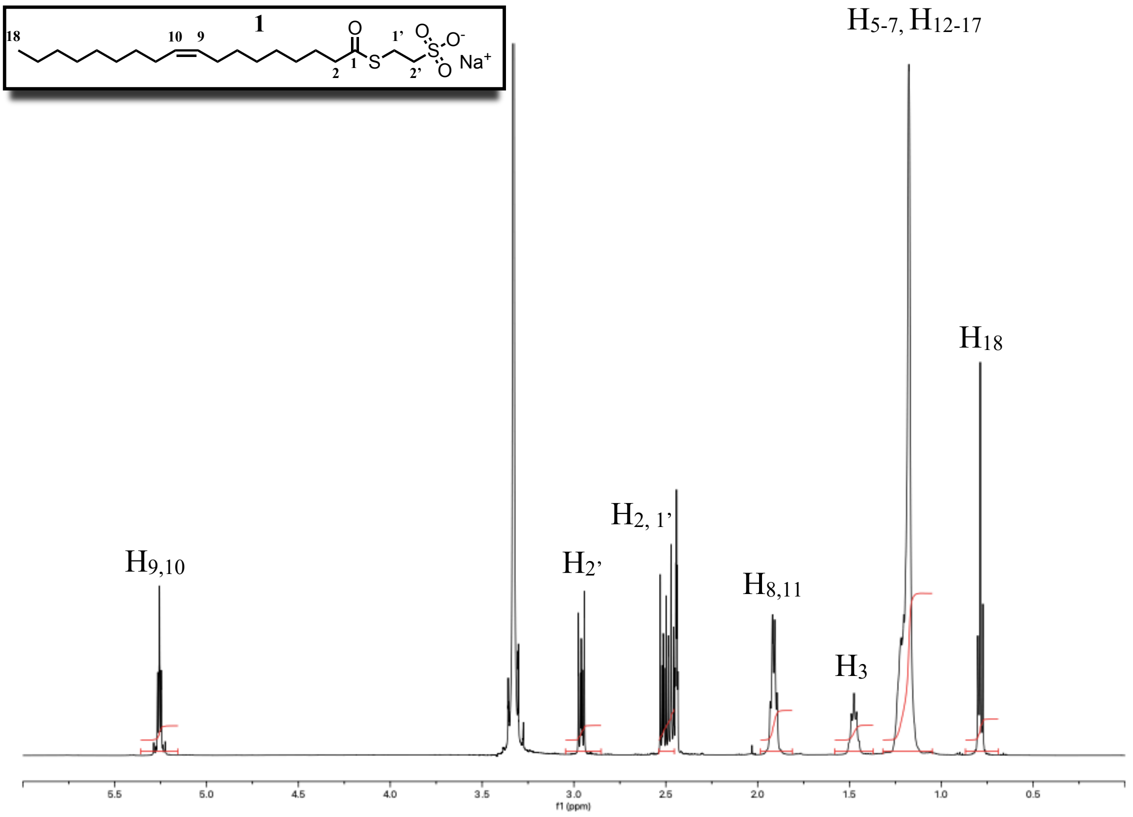

^{1}H-NMR spectrum of MESNA thiooleate **1** (d_6-DMSO, 500.13 MHz)

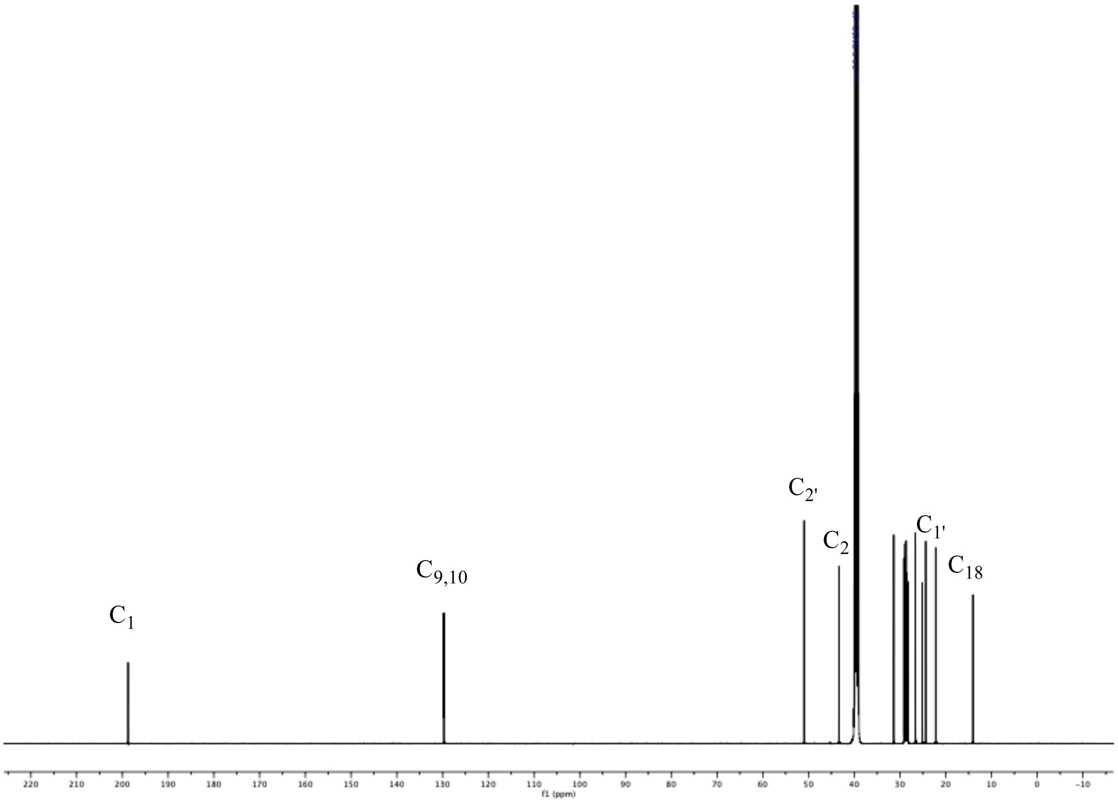

^{13}C-NMR spectrum of MESNA thiooleate **1** (d_6-DMSO, 125.77 MHz)

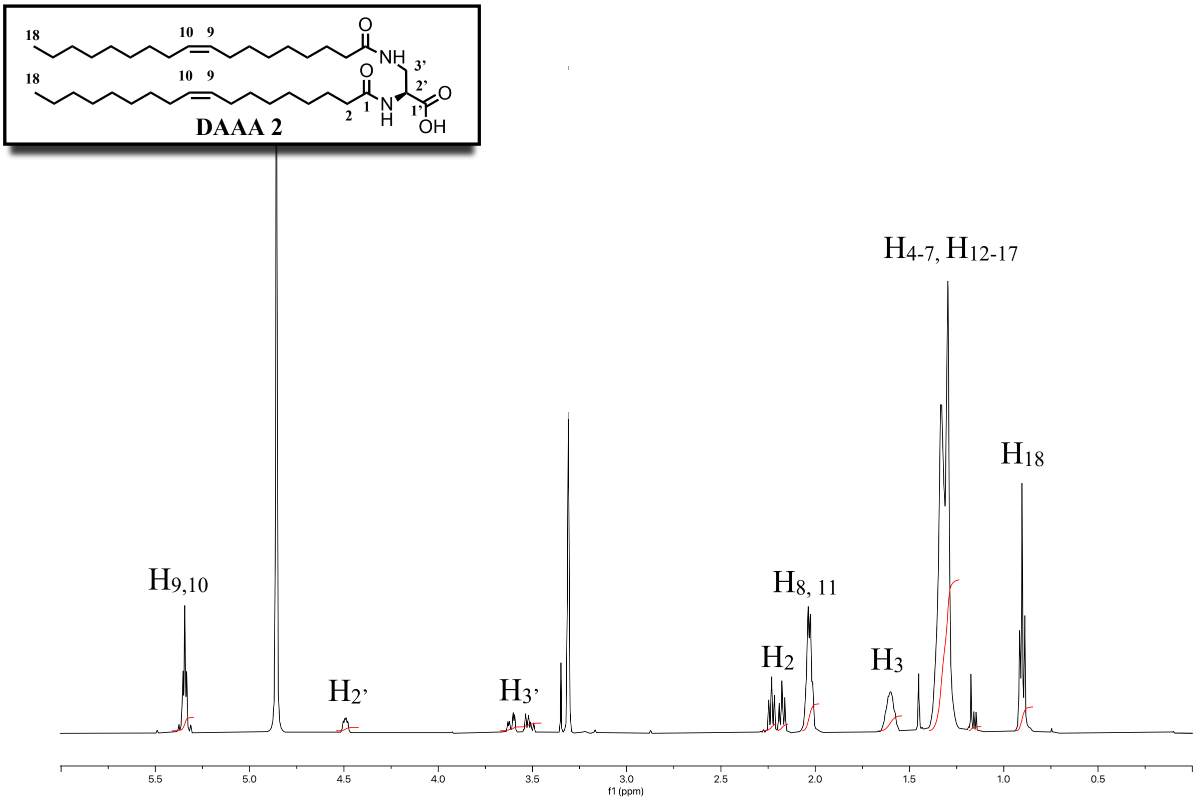

^{1}H-NMR spectrum of DAAA **2** (CD_3OD, 500.13 MHz)

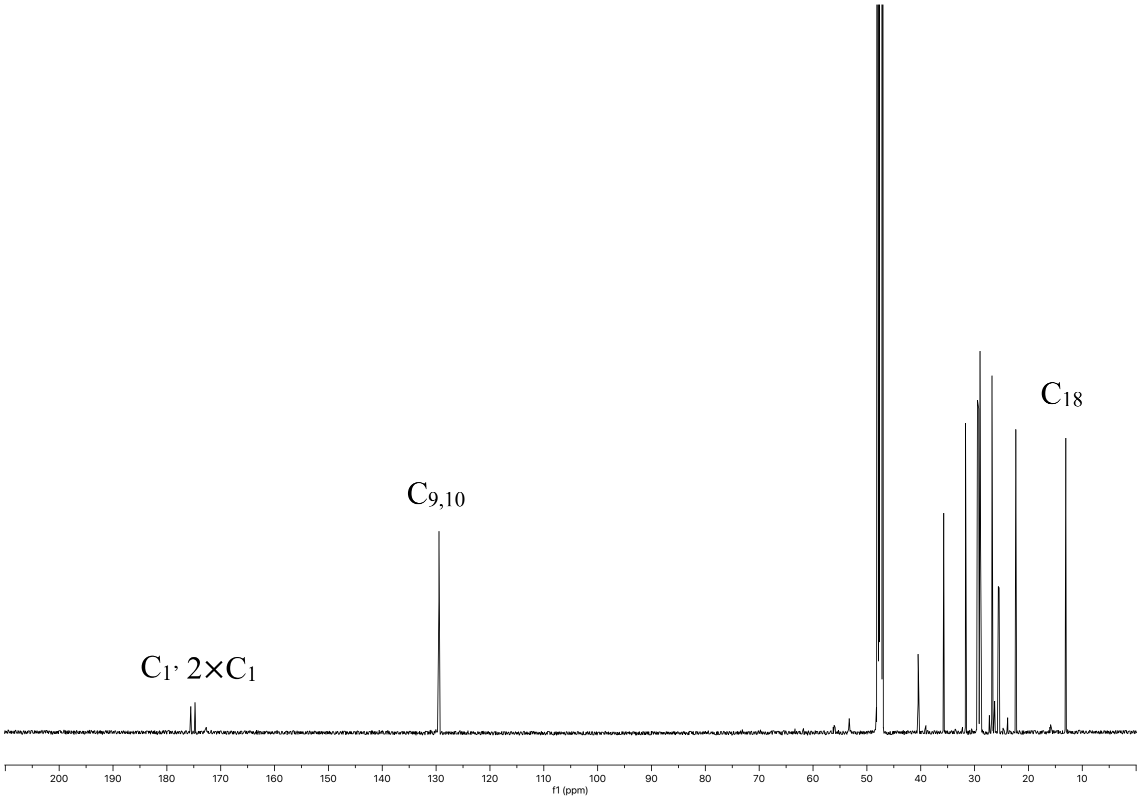

^{13}C-NMR spectrum of DAAA **2** (CD_3OD, 125.77 MHz)

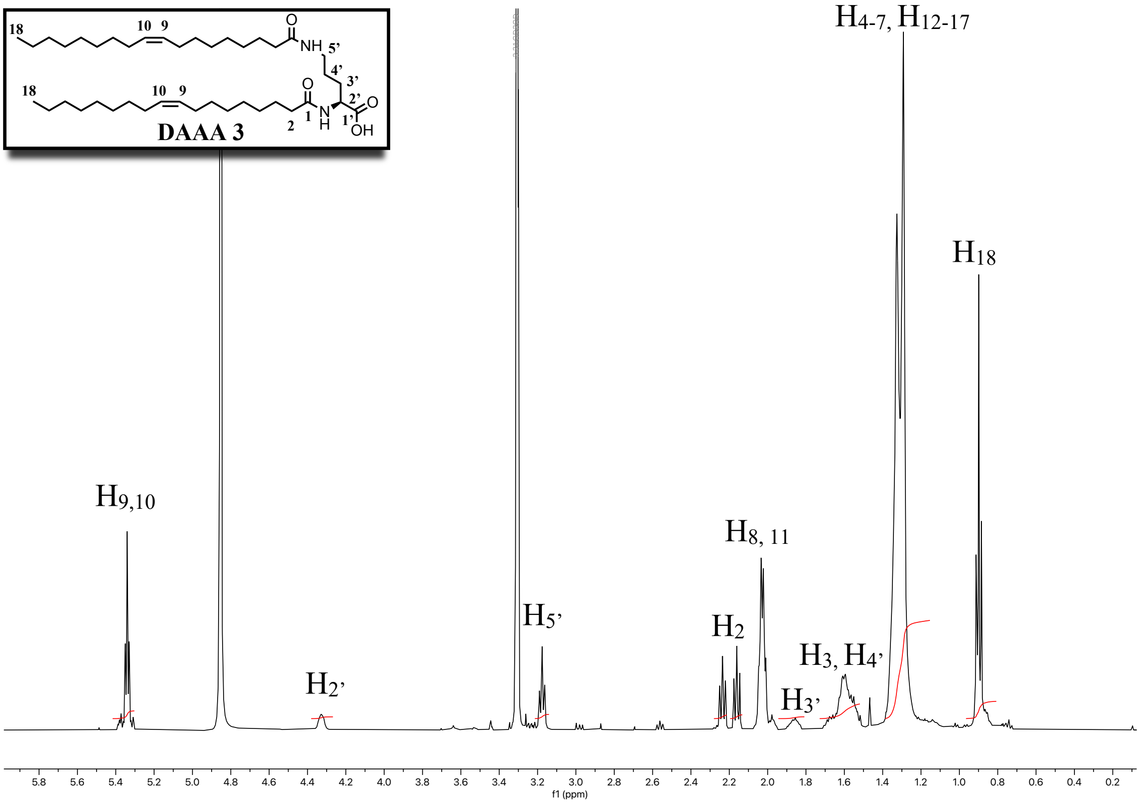

^{1}H-NMR spectrum of DAAA **3** (CD_3OD, 500.13 MHz)

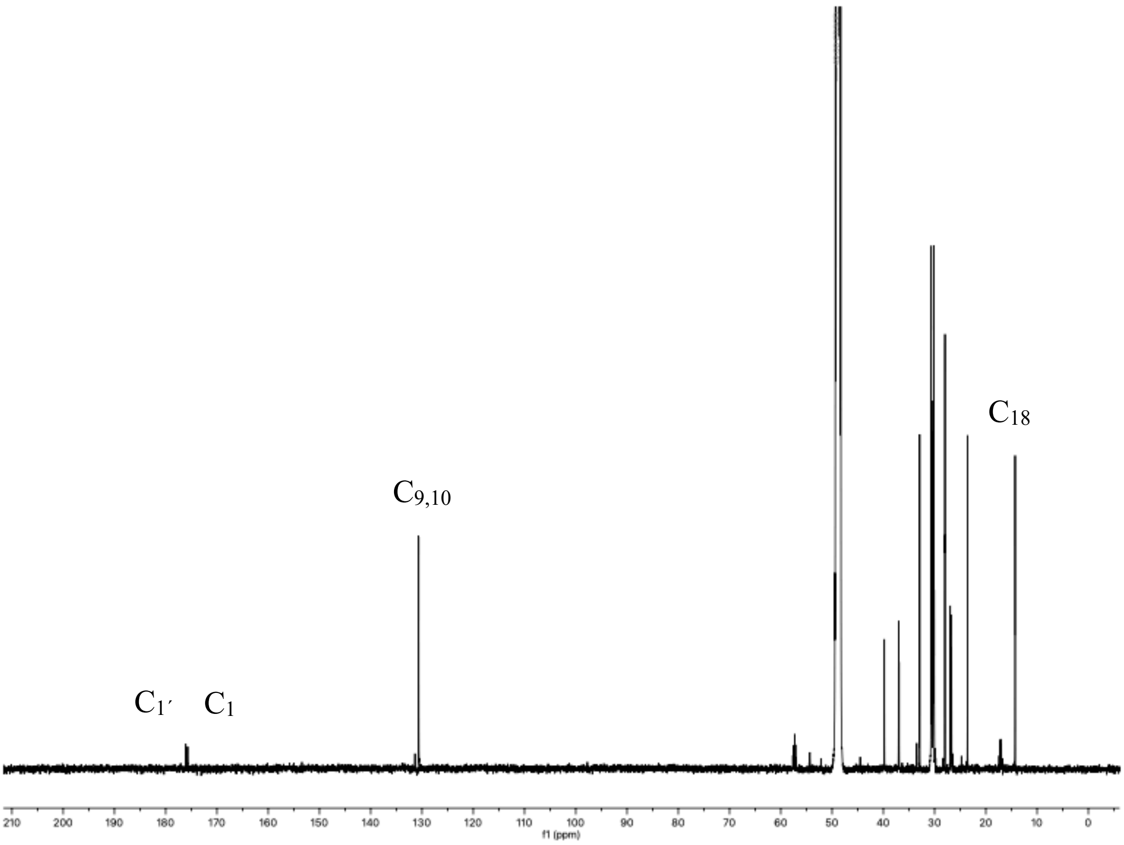

^{13}C-NMR spectrum of DAAA **3** (CD_3OD, 125.77 MHz)

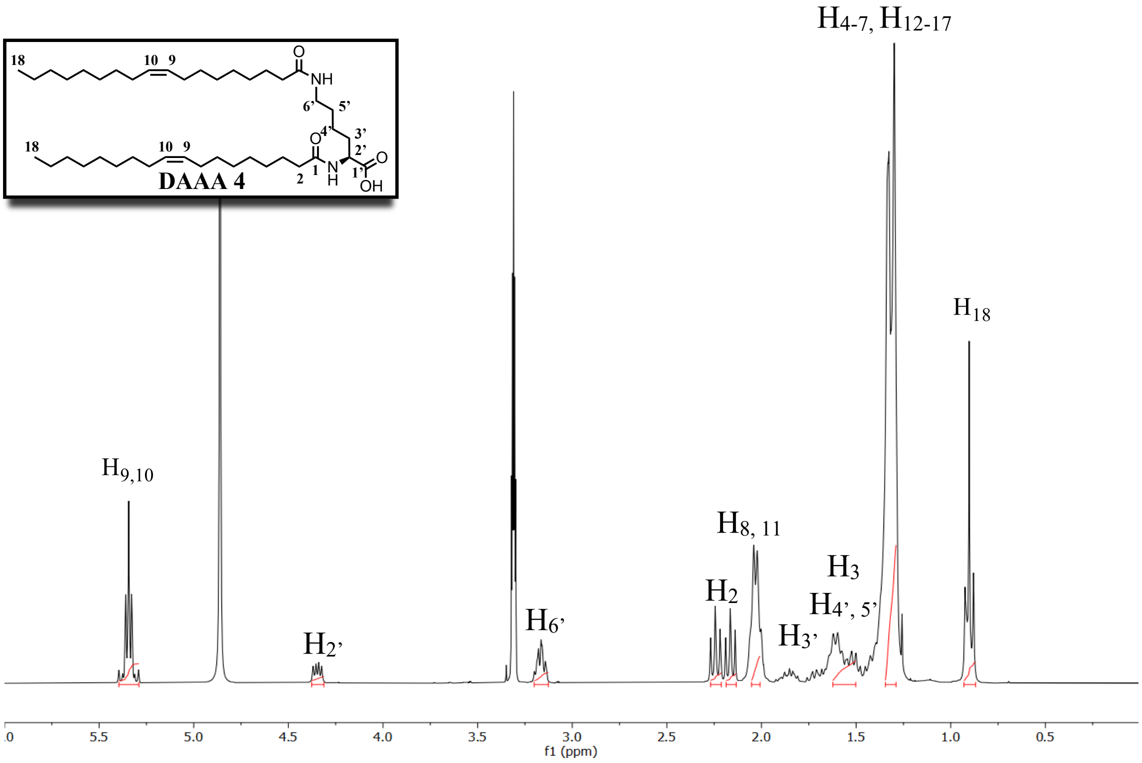

^{1}H-NMR spectrum of DAAA **4** (CD_3OD, 500.13 MHz)

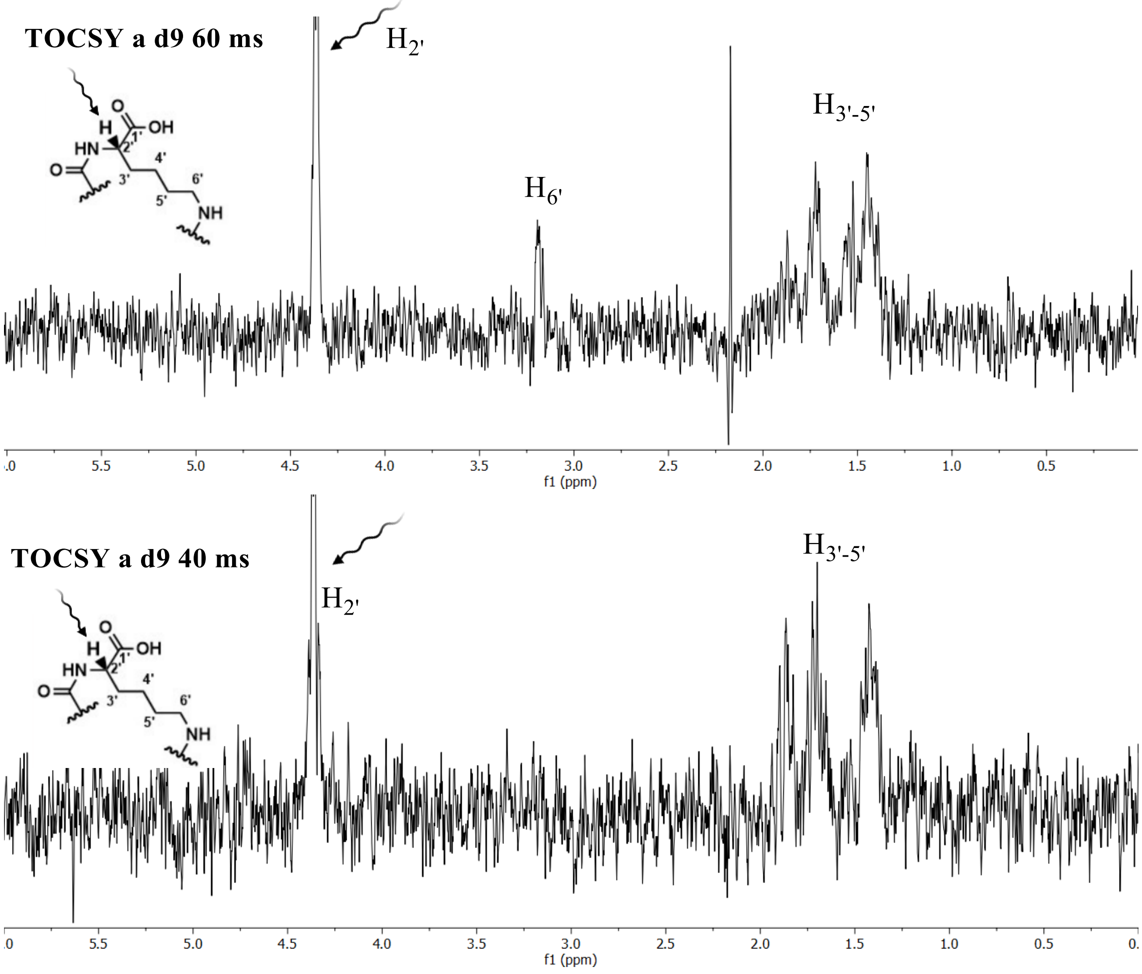

1D-TOCSY (selective proton irradiation at δ 4.41 ppm) of DAAA **4** (CD_3OD, 500.13 MHz)

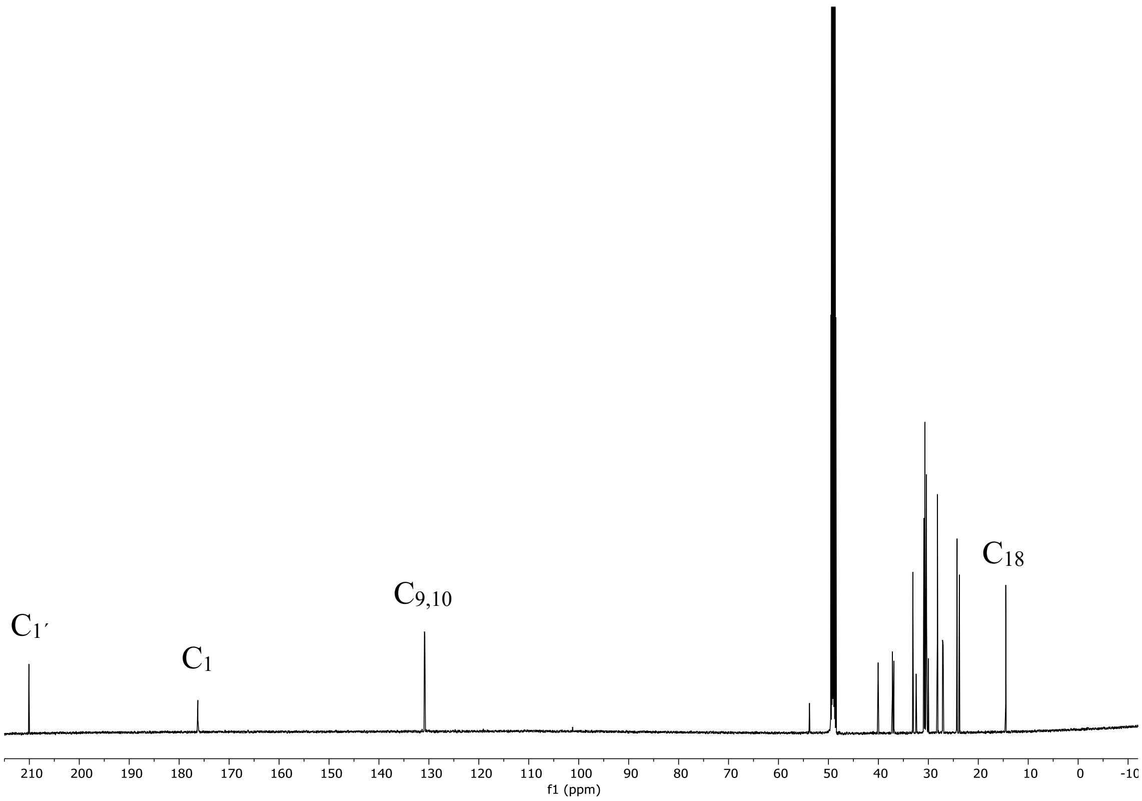

^{13}C-NMR spectrum of DAAA **4** (CD_3OD, 125.77 MHz)

Mass Spectra

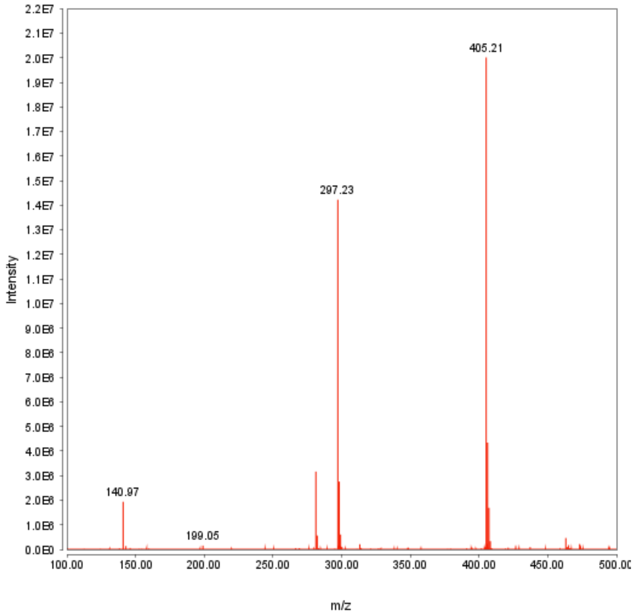

Mass spectrum of MESNA thiooleate (**1**)

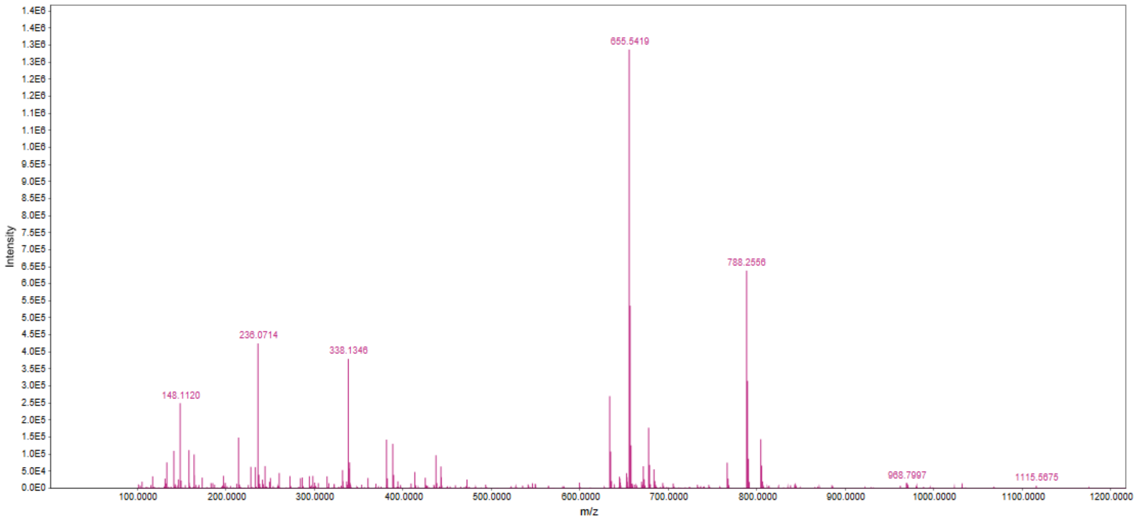

Mass spectrum of DAAA **2**.

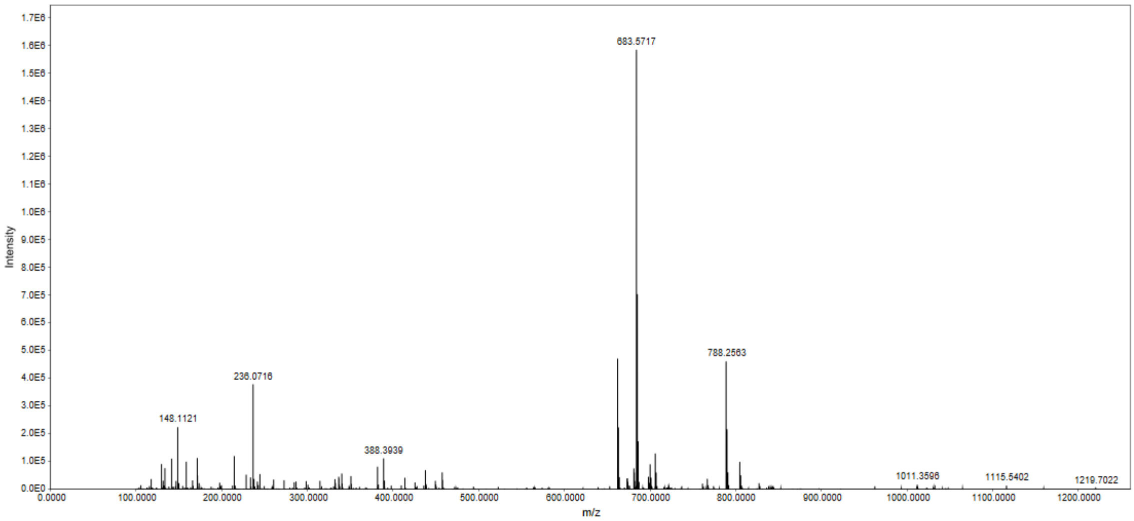

Mass spectrum of DAAA **3**.

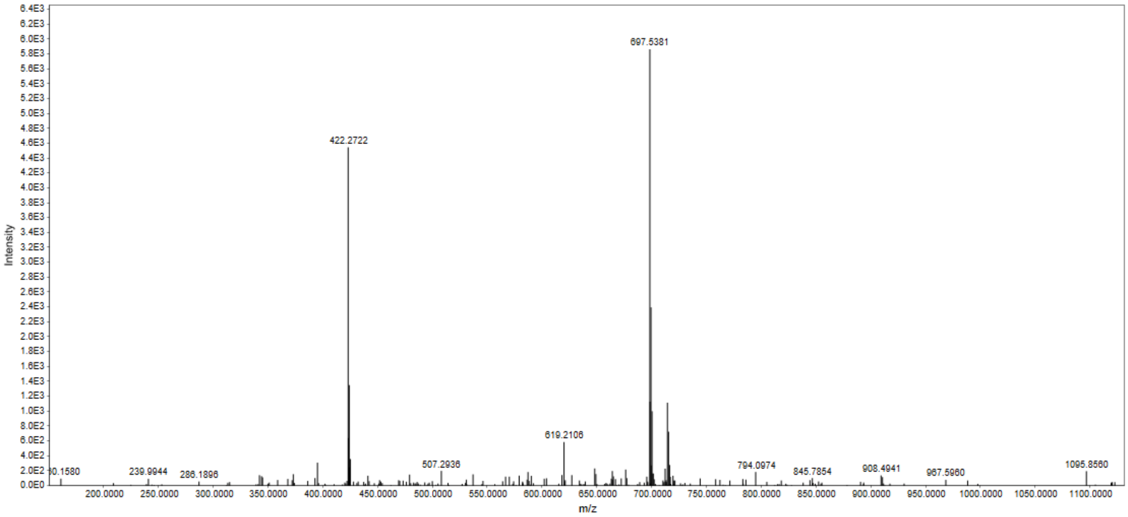

Mass spectrum of DAAA **4**.
